# mindLAMPVis as a Co-Designed Clinician-Facing Data Visualization Portal to Integrate Clinical Observations From Digital Phenotyping in Schizophrenia: User-Centered Design Process and Pilot Implementation

**DOI:** 10.2196/70073

**Published:** 2025-06-10

**Authors:** Karthik Sama, Jaya Sreevalsan-Nair, Soumya Choudhary, Srilakshmi Nagendra, Preethi V Reddy, Asher Cohen, Urvakhsh Meherwan Mehta, John Torous

**Affiliations:** 1 Graphics-Visualization-Computing Lab, International Institute of Information Technology Bangalore (IIIT-B), 26/C Electronics City, Hosur Road, Bangalore, 560100, India, 91 80 4140 7777, 91 80 4140 7704; 2 National Institute of Mental Health and Neuro Sciences, Bangalore, India; 3 Beth Israel Deaconess Medical Center, Harvard Medical School, Boston, MA, United States; 4 Consciousness Studies Program, School of Humanities, National Institute of Advanced Studies, Bangalore, India

**Keywords:** co-design, data visualization, browser-based dashboard, multivariate data mining, dimensionality reduction, local technology adoption, digital phenotyping, schizophrenia, anomaly detection, mobile phone

## Abstract

**Background:**

The potential of digital mental health to transform care delivery in low- and middle-income countries is well established. However, there remains the need to clinically and organically adapt current tools to local needs. This paper explores the process of creating a novel data visualization system for a digital mental health app and outlines the necessary steps in the process. This work demonstrates co-design involving collaboration between teams across geographies and disciplines based on clinicians’ requirements.

**Objective:**

This study aims to co-design a visualization dashboard app for clinicians through a design study with a multidisciplinary team consisting of clinicians in Boston and Bangalore, mindLAMP software developers in Boston, and computer scientists with visualization expertise in Bangalore. The app is designed to visualize derivatives of both active and passive data of patients with schizophrenia to support the research contexts of digital psychiatry clinics in India.

**Methods:**

The mindLAMP app, already used in many countries today, is adapted to offer a new clinician-facing data visualization portal, mindLAMPVis. The novel web-based portal is designed to improve clinical integration for use in India. After building the new portal, the insights from this new portal are corroborated with known clinical observations of relapse using comparative visualization. The data were taken from the mindLAMP app and processed using multivariate analysis and dimensionality reduction to make it easy and manageable for clinicians to analyze. These techniques are integrated in mindLAMPVis, thus making it a locally co-designed, developed, and deployed tool. A feasibility study of the pilot implementation of the app was completed through a domain expert study with clinician-driven case studies.

**Results:**

To assess the system, we preloaded data from 24 patients with schizophrenia, including those with relapses. Through case examples focusing on relapse risk prediction in schizophrenia, mindLAMPVis is used to identify different visualization methods to compare different analytical results for each patient. In partnership with clinicians for co-designing the app, we explored the feasibility of a comparative visualization tool for discovering patterns across different time stamps for a single patient or any patterns across patients related to the relapse episode. As an example of reverse translation, mindLAMPVis offers new features that complement the original features of mindLAMP, highlighting the mutual benefit of software adaptation and collaborative design.

**Conclusions:**

mindLAMPVis is a tailored tool designed for use in India, but it can aid in identifying and comparing behavioral patterns that may indicate clinical risk for patients in any country. mindLAMPVis offers an example of how, through technical design, feedback, and real-world clinical testing, it is feasible to adapt current software tools to meet local needs and even exceed the use cases of the original technology. mindLAMPVis also successfully incorporates both active and passive digital phenotyping data.

## Introduction

Despite the well-known potential of digital mental health to advance psychiatric care, a lack of clinical integration has hampered meaningful use [1]. Low- and middle-income countries (LMICs) are ideal settings for these technologies to help advance care and reduce access barriers. However, many of the digital tools such as apps need not be designed to cater to the clinicians’ needs [2] or locally developed [3], which affects the adoption of the technology. At the same time, this need not be a barrier as digital technologies can be easily and inexpensively customized with collaborative efforts. This paper explores one example of such tailoring by focusing on the use of the open-source mindLAMP smartphone app [4,5], where clinicians and computer scientists co-design the visualization portal that is integrable with the mindLAMP app.

Schizophrenia is considered an area of less innovation, often due to the complexity of the symptoms. However, the actual evidence supports numerous advances in digital health [6]. Aspects of the illness, such as relapse, continue to be hard for clinicians, family members, and patients themselves to predict [7]. Hence, better risk assessment and management of relapses depend on continuous monitoring and timely interventions. Recent technological developments have enabled the collection of large-scale datasets of passive (sensors) and active (surveys) data through smartphones. The availability of such data provides opportunities for understanding and predicting symptom relapses, with positive results shown across several studies [5,8]. However, the high volume, complexity, and dense time series of the data collected, along with the requirement to maintain a high level of data privacy, present significant challenges in its interpretation and clinical application.

Effective data visualization transforms this raw, large-scale data into meaningful insights to be consumed by both clinicians and patients. It is also critical for ethical considerations around digital data capture and the use of patients’ data [9]. The visual representation of data in a clear and comprehensible manner helps in identifying patterns, trends, and anomalies that may not be observable through other data analysis methods [10], referred to as data mining. Recent work shows the potential of transforming data to new formats, such as networks, and performing network analysis and visualization to derive cluster-based inferences from large-scale patient datasets [11]. Similarly, the use of deep learning models and visualization helps in predicting clinical risks from electronic health record datasets [12]. In the same vein, data mining and visualization are thus important in the case of schizophrenia, where understanding behavioral patterns and predicting relapse significantly impact patient outcomes.

The current literature strongly supports the notion that data visualizations have the potential to improve clinicians’ engagement and effectively communicate trends in patient data [13,14]. Personal health care data visualizations improve health literacy [15], enhance communication between patients and health care providers, support informed health care decisions, and enable quicker data comprehension [13,16]. Furthermore, effective visualizations increase users’ trust and willingness to share their tracking data through transparency in information usage and its integration into their health care. The use of a dashboard framework for disease surveillance is viable for effective communication of data trends in the population [17]. A recent study shows that, for patients with conditions of depression, epilepsy, and multiple sclerosis, their own choice of visualizations of their personal health data collected using wearables improves self-management of their conditions [18].

Despite these benefits concerning patient data in general, the unique nature of digital phenotyping data has led to limited efforts in its visualizations. Previous works show the role of visualizations in aiding clinicians to understand patients’ health-related behavior from digital phenotyping data [19,20]. However, such studies are limited to the analysis of passive data of patient behavior (such as physical location and smartphone usage patterns) by health care providers [21,22]. Such studies also do not investigate the integrability of such analysis to the data management framework. Although its potential for real-time analysis of passive data is being explored, visualizations of active data (such as surveys) enhance the explainability of observations in the retrospective analysis of symptom relapse cases. Given that there is no simple or universal rule for visualizations and limited collective experiences of patients and clinicians with visualizations of mental health care data, there is a clear need for expanded research of novel or uncharted methods and strategies.

In this study, we propose mindLAMPVis, which is a web-based visualization tool designed to enhance the usability of data processed through the mindLAMP platform. The data are prepared for visualization using multivariate data mining through dimensionality reduction. The inspiration for the tool derives from the request and needs of local clinicians in Bangalore, India, after initial work with the mindLAMP platform for a research study, which was developed in Boston, United States. However, the need for web-based visualization is universal, and the use of this tool is global. One of the key challenges with data collection in digital phenotyping is the lack of consistency of data recording by the patient, which often leads to insufficient data for further analysis. Comparative analysis of the patient’s data over time or patterns across different patients helps clinicians to confirm the analytical results with known clinical observations of symptom relapse and visually share or discuss with patients. Comparative visualizations in the new mindLAMPVis software enable such confirmations. Through case studies, we demonstrate how an exploratory tool, such as mindLAMPVis, identifies and compares behavioral patterns, which helps psychiatrists and other domain experts to offer more data-driven, personalized, and shared decision-making-based care.

Our research aims to gain actionable insights into raw data by integrating advanced data mining and visualization techniques into clinical practice. The relevance of such a tool lies in the global significance of digital phenotyping through mindLAMP. We hypothesize that a comparative visualization tool improves the understanding and management of schizophrenia, thereby supporting more effective, timely interventions and fostering a better provider-patient relationship. The findings from our study lead to continued co-design and refinement of data visualizations, ensuring their accessibility to both patients and clinicians. A deep dive into the state of the art of comparative visualization and methods is given in the Multimedia Appendix 1. The pilot implementation of mindLAMPVis is available in a publicly accessible website [23]. The source code for the app with a dummy input dataset is also available in a publicly accessible website [24].

## Methods

### Study Design

Our proposed clinician-facing dashboard, mindLAMPVis, is intended to compare different visualizations of the same patient. For exploratory analysis, the same visualization of different patients can be compared. Our goal was to co-design the app with clinicians, visualization experts, and software developers in Bangalore and Boston to suit the local clinical needs in LMIC.

### Co-Design Process

The dashboard is an outcome of the co-design process, that is, a collaborative design process, involving clinicians in Bangalore and Boston, mindLAMP developers in Boston, and computer scientists with visualization expertise from Bangalore. It is well understood that the usability and creativity differences between different groups must be addressed through a user-led design process [17]. The initial discussions of the multidisciplinary team arrived at the following requirements and design principles:

- The data collected on the mindLAMP app were already processed for further consumption, and the motivation for developing the dashboard was to visualize the underused derived data, such as home time, survey responses, etc.
- Clinicians are particularly interested in exploring the occurrence of phenomena with sparse data, such as relapse among individuals with schizophrenia. Data of such events tend to be sparse, owing to the difficulties in measurements of unpredictable events by the clinician and reliance on patient self-reports. Despite these challenges, such events are critical for clinicians to understand the disease progression in patients. Therefore, the team established the need for a visualization tool for research by clinicians that precedes clinical use.
- The clinicians clarified the local needs for such a tool. In a busy clinic in the Indian setting, mental health professionals would need quick access to meaningful and actionable digital behaviors obtained via the data generated from the mindLAMP app used by patients. A clinician-facing dashboard should be designed to bridge this gap by providing visual representations of complex high-dimensional data. These visualizations must first be interpretable by the clinicians and, optionally, by the patient. This serves as a stepping stone toward establishing digital psychiatry clinics in India, which are expected to experience high patient volume.
- Since the visualization is intended to be exploratory rather than confirmatory, it was decided that the portal would feature comparative visualizations. This is because comparative visualizations enable a better understanding of the data through exploration.

We followed the design study methodology (DSM) used for visualization projects [25]. DSM prescribes a 9-stage workflow for implementing visualization projects in real-world scenarios. This 9-stage framework prominently features the collaboration or co-design of domain experts and visualization researchers. The 9 sequential stages are “learn,” “winnow,” “cast,” “discover,” “design,” “implement,” “deploy,” “reflect,” and “write,” where these stages are also interlinked owing to the iterative nature of the design study. These stages are grouped into 3 top-level phases, namely, “precondition,” “core,” and “analysis,” which correspond to personal, inward-facing, and outward-facing validations, respectively. The description of the 9 stages is given in the Multimedia Appendix 1.

Here, we describe how the phases progressed in our work.

1. The precondition phase, comprising of stages from “learn” to “cast,” was implemented quicker compared to similar projects owing to the agreement of all members of the collaborative team on the mindLAMPVis specifications. The precondition phase determines the feasibility of the project, which went smoothly for our work.
2. The core phase, comprising of stages from “discover” to “deploy,” was mostly implemented by the visualization team in collaboration with the mindLAMP developer team. Owing to the physical distance between the teams and the voluntary nature of the software development process, this phase took the longest time to complete. During the “design” stage, the team narrowed down the dimensionality reduction methods and the visualizations. The “design” and “implement” stages involved back-and-forth iterations to improve on the visualizations and correct the data processing techniques based on the domain knowledge of the clinicians. During the “deploy” stage, the clinicians expressed their feedback to the entire team on the usefulness of different visualizations provided in the dashboard.
3. The analysis phase includes the “reflect” and “write” stages, which were implemented by the clinicians and the entire team, respectively. The “reflect” stage involved identifying appropriate case studies to demonstrate the usability of mindLAMPVis. In this work, the “reflect” stage involves the presentation of the usability of mindLAMPVis as a pilot implementation. A systematic study of the clinical efficacy of the visualizations is currently out of the scope of this work.

For the pilot implementation of mindLAMPVis, we have completed all the phases, except that the analysis and reflection stages have been executed only partially. In the cast stage of our work, we determined that the clinicians play the role of both designers and end users. The mindLAMP developers provided support for the existing software and access to data, and the visualization tool developers determined the data processing algorithms, visualization methods, and the actual implementation. The entire team participated in the deploy and write stage.

### Ethical Considerations

This study’s protocols were approved by the local institutional ethics committees at each of the sites for the Smartphone Health Assessment for Relapse Prevention (SHARP) project [5]. All participants provided written informed consent. Only preprocessed, deidentified data were used in the analysis. All study participants received compensation for travel and refreshments during each of the study visits. In this study, we have used the anonymized data collected from patients with a schizophrenia diagnosis from the SHARP project.

### Clinical Setting

This was a 12-month multisite, longitudinal observational study where individuals with and without schizophrenia used the mindLAMP application for active and passive data collection alongside monthly clinical evaluations for symptom relapse and cognitive performance. Patients were recruited from outpatient hospital settings if they had a diagnosis of schizophrenia based on DSM-5 criteria as ascertained by a trained psychiatrist.

### Dataset

We preloaded data from 24 patients with a schizophrenia diagnosis from the SHARP project [5]. The patients were recruited at the National Institute of Mental Health and Neurosciences, Bangalore. We used subsets of data from both active and passive data for this feasibility study. Active data include 6 surveys (see details in the Multimedia Appendix 1), and passive data include GPS and accelerometer information. In mindLAMPVis, we use the active data and the significant locations computed from the GPS locations [26]. Survey data was collected as responses to a questionnaire, and we use the data for 5 questionnaires, namely, mood, sleep, social, anxiety, and psychosis. A detailed description of the entire dataset and the subset we used is given in the Multimedia Appendix 1.

### Imputation

Missing data is inherent to digital phenotyping. Our tool supports two types of imputation methods to fill in the missing values in the data, namely, (1) last observation carried forward (LOCF) and (2) Multiple Imputation by Chained Equations (MICEs).

LOCF repeats the last observed value until the item where data are available. This method has the least computational cost and the least accuracy. While LOCF is the third most used imputation method, multiple imputation methods are considered more efficient. Multiple imputation uses different simulation models to provide multiple values for the missing data instance. MICE assumes that a multivariate distribution can be used for the incomplete variable with missing data [27]. Thus, in this method, missing values are filled using a distribution fitted to the data and are more accurate. The working of these algorithms is described in detail in the Multimedia Appendix 1.

### Dimensionality Reduction

We analyze the responses to 5 survey sections, namely, mood, sleep, anxiety, social, and psychosis factors [25]. To visualize the data in each survey section, we use multiple correspondence analysis (MCA) for dimensionality reduction, where we treat the questions in each survey section as the dimensionality of the section. The responses to each section are represented as a vector to implement dimensionality reduction.

If the time-series data for each section have a large variance in 1 or 2 components in MCA, we can conclude that the patient responses are consistent over time. This is checked using the first and second eigengaps where a high value of eigengap indicates that the responses to the questions in the specific survey section have been highly consistent. The lower-dimensional vectors and derived data are then used for visualizations.

### Clustering and Coassociation Matrix

We also cluster dates of data availability within a chosen period to check if the responses to the questions indicate unique behavioral patterns in the patient. We use the vector format of the responses for clustering in 2 different ways, namely, as an aggregate vector (AV) and a complete vector (CV). The length of the AV is that of the number of sections, and the vector component is the average value of the responses in the section. However, the CV retains all the information by concatenating the vectors for each section, thus forming a long vector. Thus, the length of the CV is the total number of questions in the survey. The AV or CV is the representation of the mental state of the patient for a specific date.

In data mining, one of the key influencing factors for the implementation of clustering is the number of clusters. The cluster count can be either given as user input or implicitly determined from the data itself. We refer to the clustering methodologies as predefined clustering and natural clustering, respectively. The former determines *k* clusters for the user-defined value of *k* based on prior knowledge, while the latter algorithmically determines the natural partition available in the data.

In cases where there is no ground truth or baseline solutions in data mining, we use the consensus of outcomes from several methods, thus establishing strength in numbers. The consensus in the clustering of items is represented in the form of a matrix, namely, the coassociation matrix. This matrix represents how likely any 2 items of the entire set are to be present in the same cluster. If they are highly similar, then their coassociation value is 1, and if highly dissimilar, then it is 0. Thus, the coassociation between any 2 dates in our case is represented as a ratio between 0 and 1.

More details on the clustering and coassociation matrix are given in the Multimedia Appendix 1.

### Visualizations

**Figure 1.**
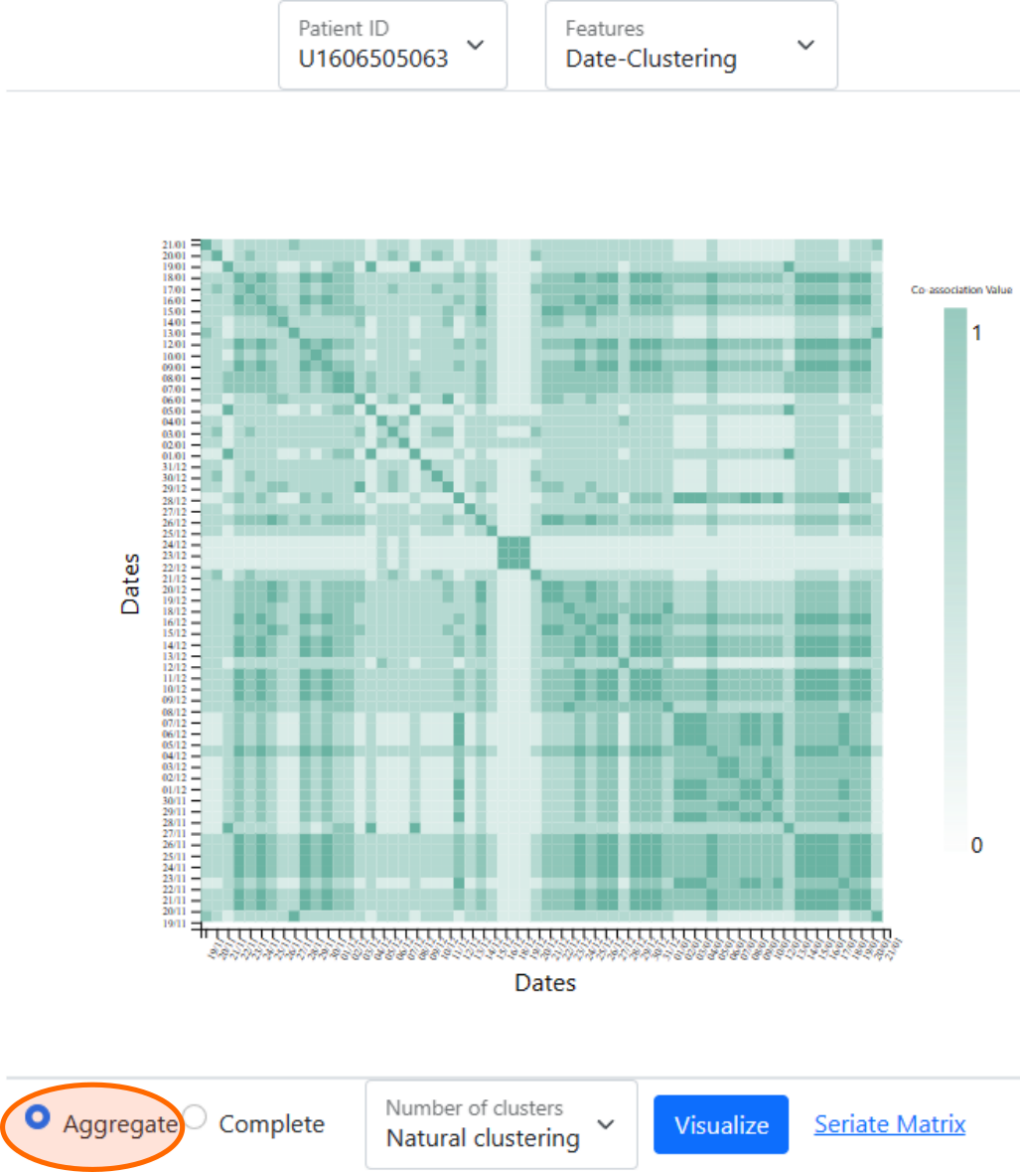
Matrix visualization of the consensus matrix of clustering of calendar dates for chosen aggregation and clustering strategies, for a selected patient. The orange highlight shows the choice of AV for this example. This chart is generated from the anonymized data of a patient from the Bangalore cohort collected during 2021-2022 in the SHARP project [5]. AV: aggregate vector; SHARP: Smartphone Health Assessment for Relapse Prevention.

**Figure 2.**
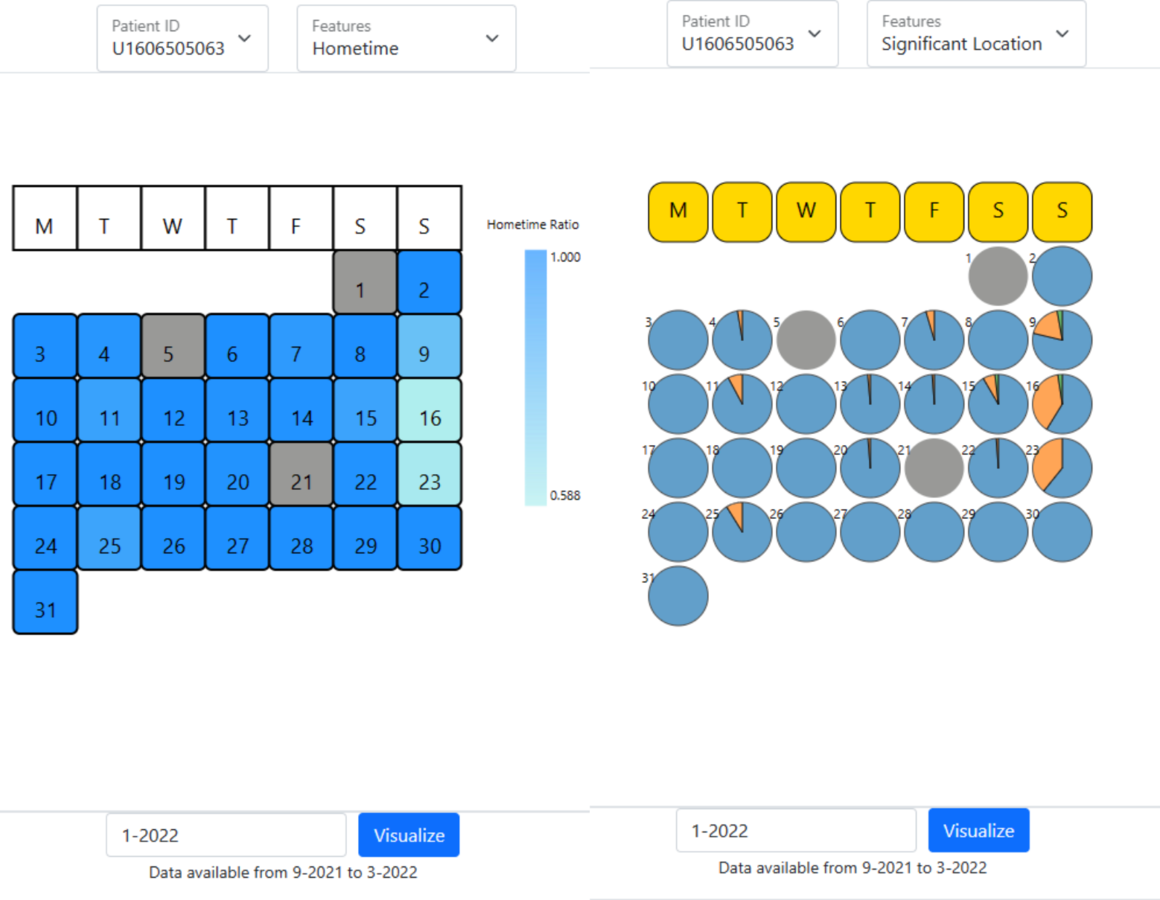
Patient-specific calendar view visualizations of (left) home time (V1P) showing the percentage of home time colored using a gradient color palette, (right) the distribution of significant locations (V2P) using pie-chart glyphs. This chart is generated from the anonymized data of a patient from the Bangalore cohort collected during 2021-2022 in the SHARP project [5]. P: passive; V: visualization; SHARP: Smartphone Health Assessment for Relapse Prevention.

In our proposed visualization tool, mindLAMPVis, we support the following visualizations (V) of active (A) and passive (P) data:

- MCA trend (V1A) of survey data (Figure S1 in Multimedia Appendix 1),
- MCA eigengap (V2A) of survey data (Figure S2 in Multimedia Appendix 1),
- Date-clustering (V3A) of survey data (Figure 1),
- Home time (V1P) of significant locations data (Figure 2),
- Significant location (V2P) of significant locations data (Figure 2).

Here, we mention the knowledge derived from each visualization, but the interpretation of the visualizations is given in detail in the Multimedia Appendix 1. V1A shows the temporal trend of the first MCA component for each survey section. V2A shows which survey section has coherent or consistent response data. V3A shows consistency of behavior over a set of dates, including periods. V1P and V2P show the amount of time spent at home (or the most significant location) and the proportion of time spent daily across significant locations. They demonstrate trends over calendar months. These 5 views enable the clinician to observe anomalous behavior in the trends a month before the relapse and during the relapse for the concerned patient. The anomalies imply inconsistencies in the behavior of the patient leading up to the relapse event.

**Figure 3.**
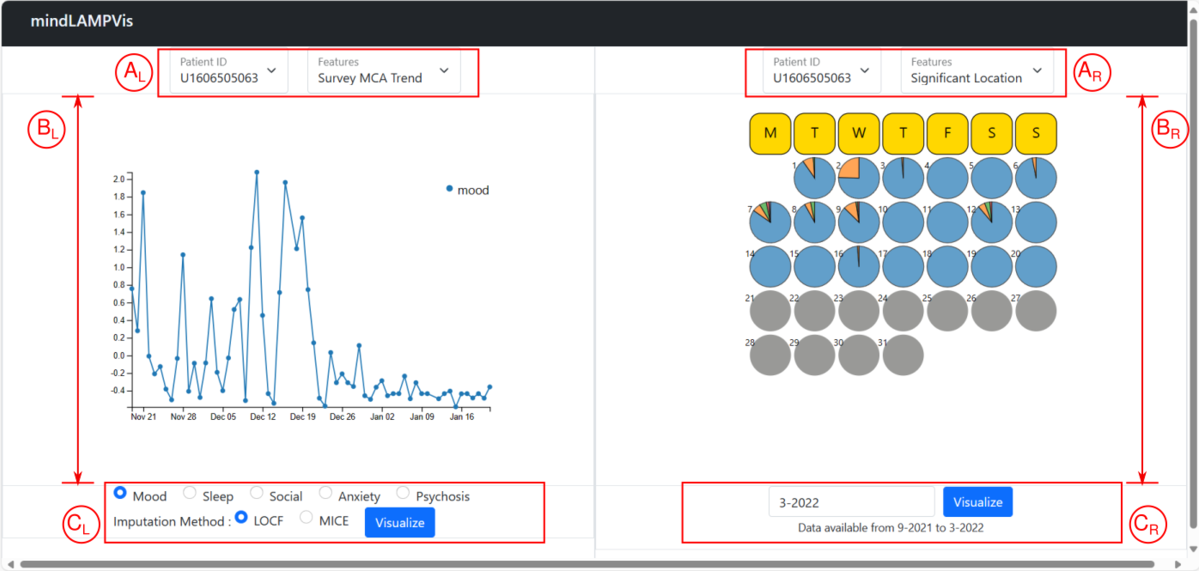
View of the 2-panel graphical user interface with annotations of its components. A, B, and C indicate feature-controls, canvas, and feature-parameter-controls, respectively, and the subscripts L and R indicate the left and right panels, respectively. The left and right panels have the same design with clean lines. These charts in the screenshot are generated from the anonymized data of a patient from the Bangalore cohort collected during 2021-2022 in the SHARP project [5]. L: left; R: right; SHARP: Smartphone Health Assessment for Relapse Prevention.

The layout of our tool is organized as a 2-panel one, along with control widgets to allow comparative visualizations (Figure 3). The checklist of the iCHECK-DH guidelines [28] for reporting this design study is present as Checklist 1.

## Results

### Case Study

This paper describes the development and feasibility of using mindLAMPVis to draw parallels in understanding individual-specific relapse signatures in patients with schizophrenia who participated in a research study in India.

**Figure 4.**
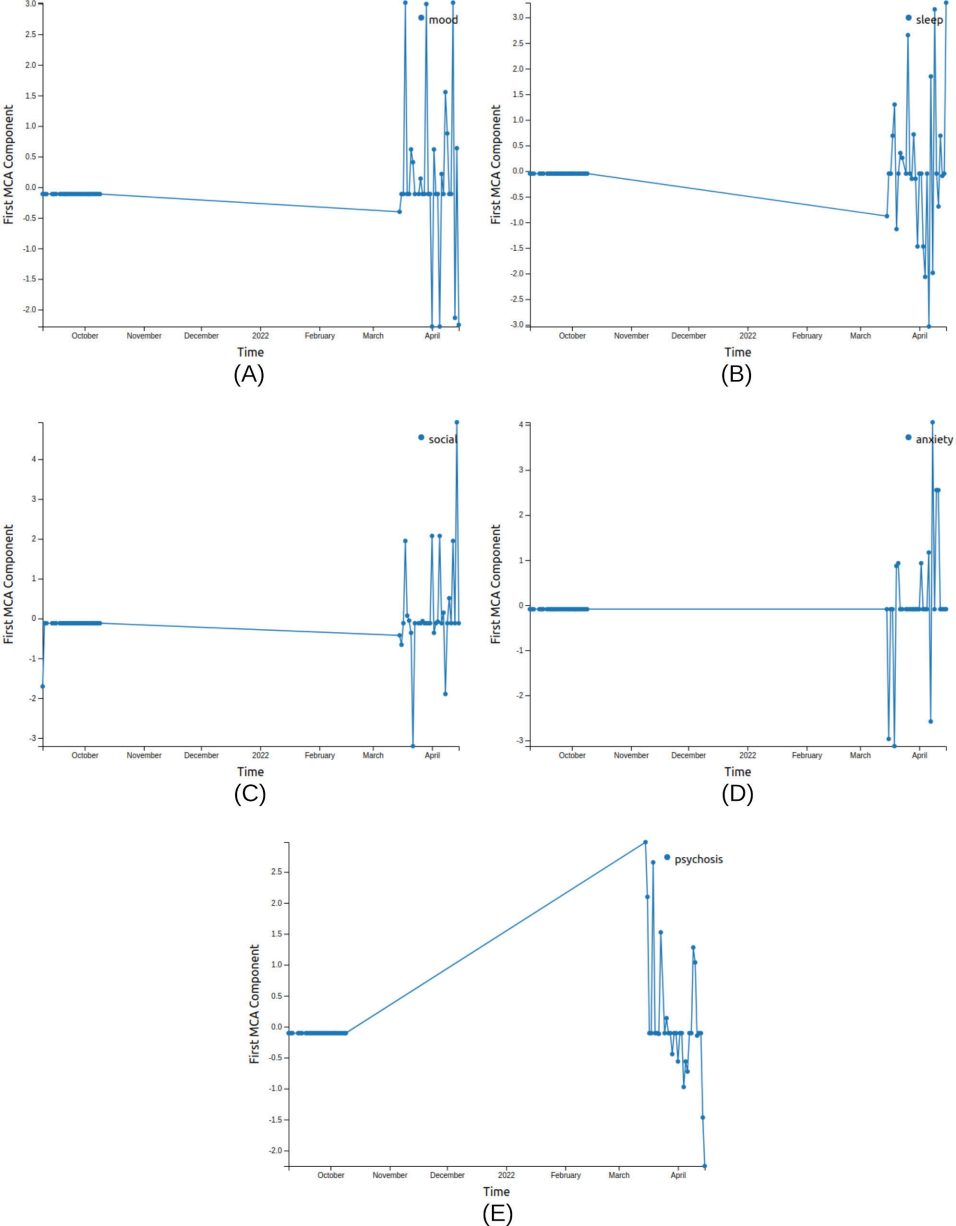
MCA trend (V1A) visualizations for patient X using MICE imputation for the 5 survey sections, namely, (A) mood, (B) sleep, (C) social, (D) anxiety, and (E) psychosis. The anomalous points with high-frequency spikes coincide within a month leading to relapse. This chart is generated from the anonymized data of the patient from the Bangalore cohort collected during 2021-2022 in the SHARP project [5]. The clinicians logged a relapse in April 2022 for this patient. A: active; V: visualization; MCA: multiple correspondence analysis; MICE: Multiple Imputation by Chained Equation; SHARP: Smartphone Health Assessment for Relapse Prevention.

The clinicians in Bangalore performed a deep dive analysis of the visualizations obtained for 2 specific patients, A and B, from mindLAMPVis. Both patients have sufficient active data for performing an analysis. Patient X had a single relapse, and patient Y had two relapses within 3 months. We compare our visualizations with those of anomaly plots [5] in Figures4^8^, which are for interpreting the passive data exclusively. The quantitative analysis of the MCA trend values for patient X (Table 1) and patient Y (Table 2) also shows interesting anomalies.

**Figure 5.**
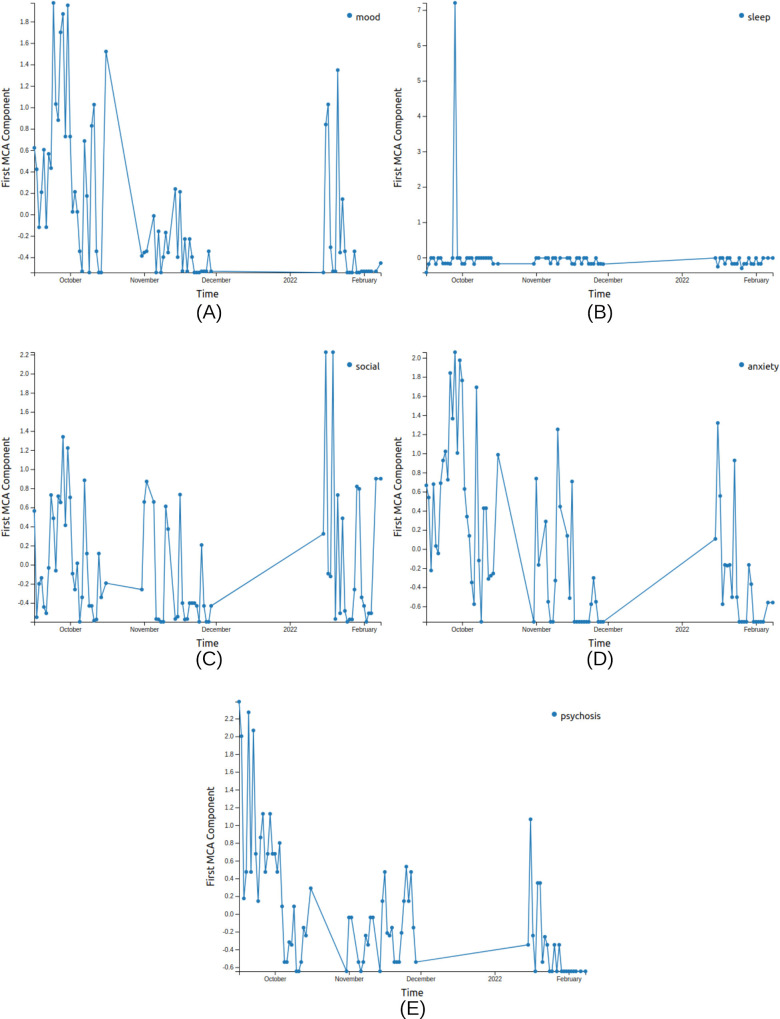
MCA trend (V1A) visualizations for patient Y using LOCF imputation for the 5 survey sections, namely, (A) mood, (B) sleep, (C) social, (D) anxiety, and (E) psychosis. The anomalous points with high-frequency spikes coincide with a month leading to relapse, and 2 relapse periods can be observed here. This chart is generated from the anonymized data of the patient from the Bangalore cohort collected during 2021-2022 in the SHARP project [5]. The clinicians logged multiple relapses in October 2021 and January 2022 for this patient. A: active; V: visualization; MCA: multiple correspondence analysis; LOCF: last observation carried forward; SHARP: Smartphone Health Assessment for Relapse Prevention.

**Figure 6.**
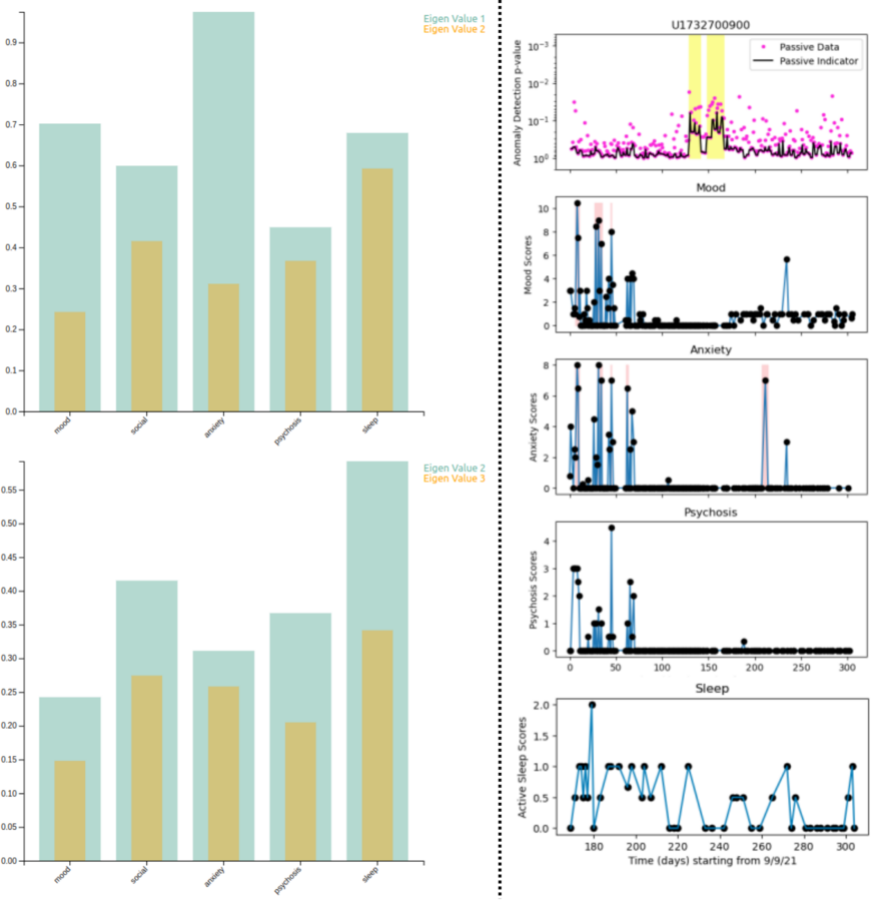
MCA eigengap (V2A) visualization for patient X using LOCF imputation for the 5 survey sections, shows significant first and second eigengaps in (left) anxiety and sleep, respectively. We observe similar patterns in (right) the anomaly plot for the same period. This chart is generated from the anonymized data of the patient from the Bangalore cohort collected during 2021-2022 in the SHARP project [5]. The clinicians logged a relapse in April 2022 for this patient. A: active; V: visualization; MCA: multiple correspondence analysis; LOCF: last observation carried forward; SHARP: Smartphone Health Assessment for Relapse Prevention.

**Figure 7.**
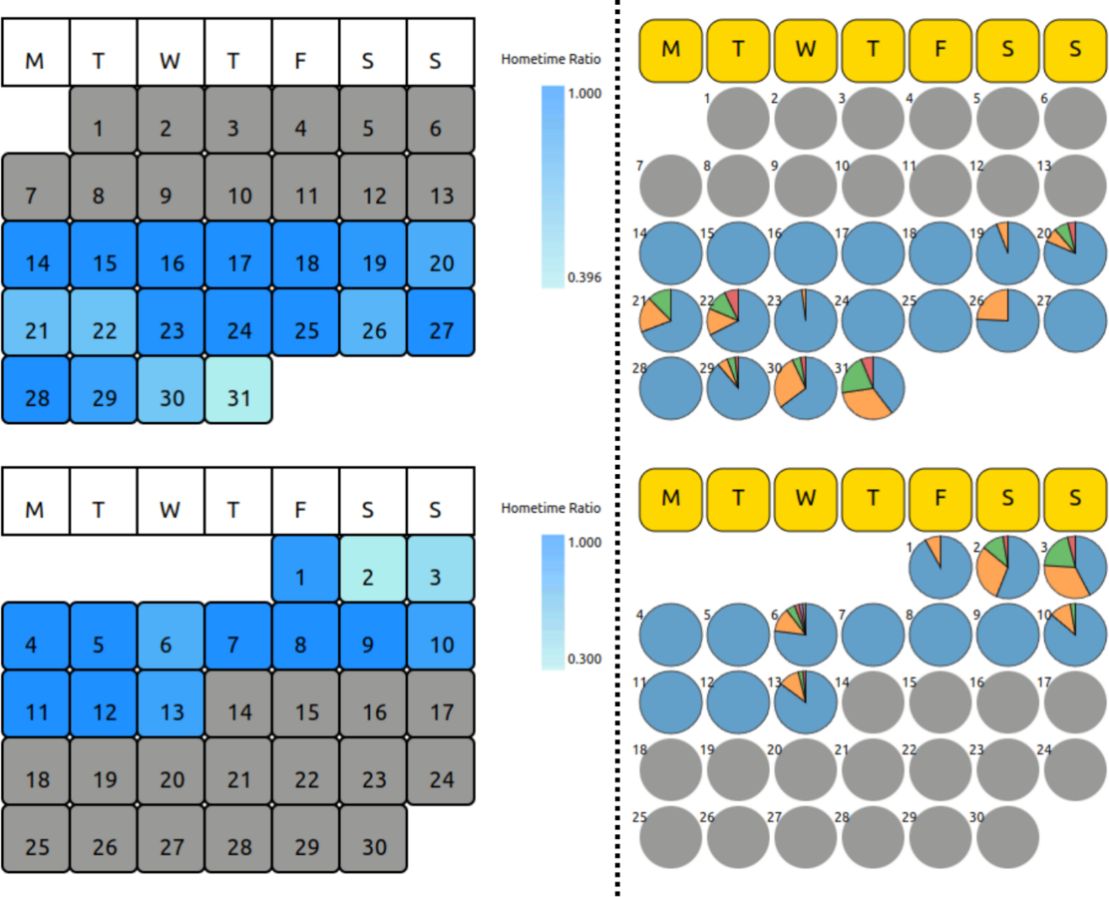
(Left) Home time (V1P) and (right) significant locations (V2P) visualizations of patient X, for the month (top) preceding and (bottom) of the relapse. This chart is generated from the anonymized data of a patient from the Bangalore cohort collected during 2021-2022 in the SHARP project [5]. The clinicians logged a relapse in April 2022 for this patient. P: passive; V: visualization; SHARP: Smartphone Health Assessment for Relapse Prevention.

**Figure 8.**
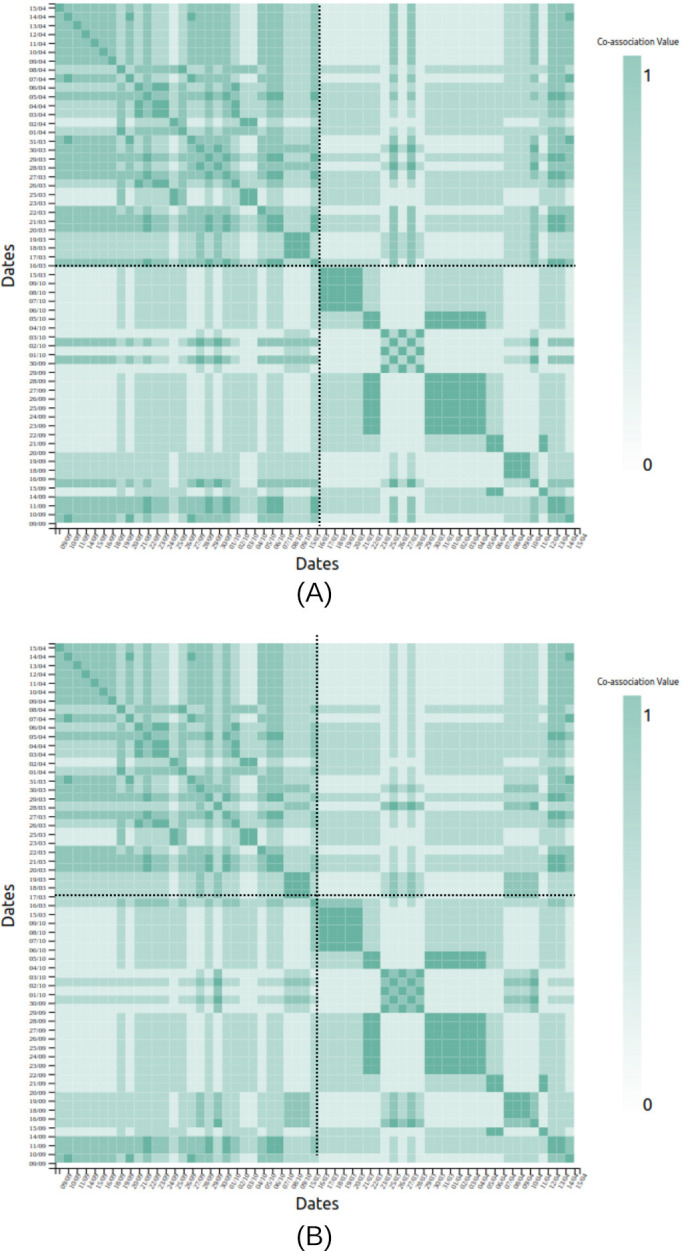
Coassociation matrix generated using (A) AV and (B) CV for NClust for patient X. The dates increment upward and rightward in the matrix, and the black dotted line marks the date marking 1 month before the relapse. This chart is generated from the anonymized data of the patient from the Bangalore cohort collected during 2021-2022 in the SHARP project [5]. The clinicians logged a relapse in April 2022 for this patient. AV: aggregated vector; CV: complete vector; NClust: natural clustering; SHARP: Smartphone Health Assessment for Relapse Prevention.

**Table 1.** MCA^a^ trend analysis for patient X using MICE^b^ imputation for the 5 survey sections, namely, mood, sleep, social, anxiety, and psychosis. This quantitative analysis of the data visualized in Figure 4 shows the mean and SD for the number of sample data points during the control period, and the month leading to the relapse date (relapse). This table is generated from the anonymized data of the patient from the Bangalore cohort collected during 2021‐2022 in the SHARP^c^ project [5]. The clinicians logged a relapse in April 2022 for this patient. For patient X, the SD is remarkably higher during the month leading to the relapse than that of the control period, for all sections.

Patient X	MCA with MICE imputation	
	Control (n=28), mean (SD)	Relapse (n=31), mean (SD)
Mood	0.13 (0)	–0.12 (1.29)
Sleep	–0.14 (0)	0.12 (1.37)
Social	0.11 (0.28)	0.1 (1.35)
Anxiety	0.14 (0)	–0.12 (1.37)
Psychosis	–0.23 (0)	0.21 (1.03)

a MCA: multiple correspondence analysis.

b MICE: Multiple Imputation by Chained Equation.

c SHARP: Smartphone Health Assessment for Relapse Prevention.

**Table 2.** MCA^a^ trend analysis for patient Y using LOCF^b^ imputation for the 5 survey sections, namely, mood, sleep, social, anxiety, and psychosis. This quantitative analysis of the data visualized in Figure 5 shows the mean and SD for the number of sample data points during the control period, the month leading to the first relapse date (relapse-1), and that to the second relapse date (relapse-2). This table is generated from the anonymized data of the patient from the Bangalore cohort collected during 2021‐2022 in the SHARP^c^ project [5]. The clinicians logged a relapse in April 2022 for this patient. For patient Y, the SD is higher during the control period than the months leading up to the relapse than that of the control period, for all sections except for social, when σ is relatively high during the second relapse.

Patient Y	MCA with LOCF imputation		
	Control (n=30), mean (SD)	Relapse-1 (n=25), mean (SD)	Relapse-2 (n=23), mean (SD)
Mood	0.51 (0.75)	–0.35 (0.22)	–0.26 (0.54)
Sleep	0.16 (1.31)	–0.09 (0.08)	–0.11 (0.1)
Social	0.07 (0.55)	–0.21 (0.5)	0.14 (0.84)
Anxiety	0.57 (0.78)	–0.36 (0.57)	–0.33 (0.56)
Psychosis	0.47 (0.85)	–0.19 (0.35)	–0.39 (0.42)

a MCA: multiple correspondence analysis.

b LOCF: last observation carried forward.

c SHARP: Smartphone Health Assessment for Relapse Prevention.

### Anomaly Plots

Using both sensor and survey data as passive and active acquisitions, respectively, for relapse prediction in psychosis is highly feasible [8]. Anomaly detection is an effective data analysis method used to identify data points that significantly deviate from the majority, indicating potential errors, unusual events, or significant changes in the observed process. These algorithms enable researchers and clinicians to longitudinally identify abnormal deviations from an individual’s typical sensor and survey data, which is indicative of relapse [5]. The anomaly plot (Figure 6) is the visual representation of potential relapse events and unusual data behavior, providing valuable insights for monitoring schizophrenia relapse. A detailed description of the interpretation of anomaly plots is given in the Multimedia Appendix 1.

### Observations

#### Patient X

We compared the MCA trend for the patient between the LOCF and MICE imputation methods for data preprocessing. We found that the MCA trend for all 5 survey sections with MICE imputation shows a characteristic anomaly in the month leading to the relapse (Figure 4). There is an uncharacteristically high SD during the month leading to relapse in all sections (Table 1), indicative of erratic behavior during that period. We also visualized the passive data using home time (V1P) and significant locations (V2P; Figure 7).

Using MCA eigengap visualization (V2A), we observe that the first eigengap is significant for anxiety, and the second eigengap is significant for sleep, which is confirmed as spikes (pink highlights) at ∼188 days in the anomaly plot for sleep and anxiety (Figure 6). These spikes occur in the anomaly plot a month before the relapse, as expected. We observe that this corresponds to the anomalies in the passive data (Figure 7).

The coassociation matrix visualization for date clustering (V3A) shows a clear cluster of dates in the month leading to the relapse for patient X (Figure 8), where we observe similar perceptible matrices for clustering using both AV and CV.

#### Patient Y

We observe that the 2 relapse periods are represented in the MCA trend visualizations (V1A) generated using LOCF imputation. The 2 periods, October-November 2021 and January-February 2022, show anomalous behavior in all survey sections except sleep (Figure 5). There is an unusually high SD observed in these MCA values in the social section in the month leading to the second relapse (Table 2), indicative of erratic social behavior during that period.

## Discussion

### Principal Findings

Through this work, we found that offering multiple visualization options (Figures1  2, and Figures S1 and S2 in Multimedia Appendix 1) for comparative analysis was the most productive approach for clinicians to derive inferences. This is evident from the clinicians’ feedback on how there is value from each visualization involving active and passive digital-phenotyping data for different use cases of schizophrenia. Throughout the process, we received feedback from software development, research, and clinical teams from Boston and Bangalore, which helped us co-design and rapidly iterate through several prototypes. Finally, as demonstrated in our case examples, we iteratively assessed and improved our tool with real-world data to ensure it offered clinical value. Through this process of technical work, design, feedback, and real-world clinical testing, we demonstrate that multidisciplinary teams can likely adapt and extend software tools as well.

Our software and results support how digital mental health approaches can be adapted and personalized to the clinical needs of local sites. They also illustrate that local solutions from LMICs are often of interest to global users and highlight the true potential of digital mental health tools. With the 2 functional and independent visualization panels in mindLAMPVis, a user can select different options for the same data, draw comparisons, and discuss findings with patients and family members. The tool explicitly allows users to select the imputation method, thus ensuring they know the choice of data preprocessing method. The 2-panel approach can also be easily extended to new conceptualizations of how to visualize existing or new data.

The feasibility of using mindLAMPVis for explorative analysis by the clinicians is the highlight of our case study. For patients X and Y (Results section), we observe a discernible change in their behaviors for a month before the relapse of their symptoms. The active data, which included the reporting of psychological symptoms such as anxiety, mood, psychosis, and sleep, clearly demonstrated this change. The passive geo-location data (GPS) also showed a significant increase in home time during the period preceding the relapse of actual psychiatric symptoms. It is interesting to note confirmatory trends across both the active and passive data streams. These findings highlight that both clinicians and patients can observe and identify patterns of individual digital behaviors and that monitoring can warn them about the early signs of relapse. By not only informing the risk of relapse but also identifying the signals related to that risk for each patient, this approach offers the opportunity to deliver both personalized and preventative care. Data science methods that offer explainability are increasingly valued over black-box models. While they may seem like a simple confirmation of previous findings, the methods used in mindLAMPVis enhance explainability in retrospective analyses. This can be critical in moving the field forward with greater transparency and interpretability.

mindLAMPVis is required for an extensive back-and-forth (ie, nonlinear study) in patient behavior analysis for a mental health illness where the risk of relapse is difficult to identify for even experienced clinicians [7,29]. This can be extended to similar challenging illnesses for which digital phenotyping enables in-depth analysis.

### Limitations

But there are still many steps. Technological advances such as mindLAMPVis also raise related questions about training clinicians to better understand the data and how it may fit into their clinical workflow. In response, our team has developed a digital navigator role to support both clinicians and patients, with aspects of the training focused on interpreting data visualizations [30]. The visualization methods in mindLAMPVis might not be immediately intuitive for most clinicians and health care workers without extra support. However, we believe that additional training is simple and will be essential for them to effectively interpret the data, particularly in light of recent advances in data collection within the health care sector. If psychiatry is to make use of novel data streams such as digital phenotyping, new tools such as mindLAMPVis must be paired with new training around how to interpret and use the outputs in care.

Beyond the technical results, our paper highlights how co-design is feasible through adapting existing software tools. While the technical challenges in working with the data required the computer science expertise of our team, the universal nature of coding enabled rapid progress. As interest in digital psychiatry expands, tools that combine data analysis (data mining) and visualization, such as mindLAMPVis, simplify the understanding and interpretation of digital behaviors for both clinicians and patients. This ensures that the work is more accessible and allows for more collaboration from all stakeholders. To date, work on this type of data visualization has been called for, with returning individual results from digital phenotyping highlighted as a critical gap in the field [31]. mindLAMPVis helps to bridge that gap and enable the next generation of more equitable advances.

### Conclusions

Through enabling data visualizations, our portal, mindLAMPVis, supplements the interpretation of data available from the mindLAMP platform by clinicians and highlights innovation from LMICs around creating solutions to global health challenges with technology. Our work demonstrates the potential of the visualizations of both active and passive data collected in digital phenotyping in schizophrenia to inform care and support preventative personalized treatments. Our case studies show how discernible patterns appeared on the visualizations a month before the relapse. The increase in the development and usage of such analytical tools reinforces the universality of the mindLAMP platform and provides new insights into the data.

## Supplementary material

10.2196/70073 Multimedia Appendix 1 Related work on visualizations and design study methodology, data processing methods, visualization methods, and the software development specifications of mindLAMPVis.

10.2196/70073 Checklist 1 iCHECK-DH guidelines for reporting the design study of mindLAMPVis. iCHECK-DH: Guidelines and Checklist for the Reporting on Digital Health Implementations.

## Abbreviations

AV: aggregate vector
CV: complete vector
DSM: design study methodology
LMIC: low- and middle-income country
LOCF: last observation carried forward
MCA: multiple correspondence analysis
MICE: Multiple Imputation by Chained Equation
SHARP: Smartphone Health Assessment for Relapse Prevention
