# Supplementary material for: mindLAMPVis as a Co-Designed Clinician-Facing Data Visualization Portal to Integrate Clinical Observations From Digital Phenotyping in Schizophrenia: User-Centered Design Process and Pilot Implementation
Source: JMIR Form Res. 2025 Jun 10;9:e70073. doi: 10.2196/70073 (PMC12173093; doi:10.2196/70073)
Supplement: Multimedia Appendix 1 [file formative-v9-e70073-s001.docx]

This multimedia appendix includes a detailed description of related work, proposed methodology including visualization methods, and the mindLAMPVis software specifications, as per design.

**Section S1. Related Work**

In this section, we review the literature on digital mental health tools, comparative visualization design, and relevant visualization techniques.

**Section S1.1. Digital Mental Health Tools**

Incorporating information technology in managing mental health conditions has increasingly become a scalable solution, and currently, there are two alternatives to this solution [1]. These solutions rely on either a monothetic, i.e., single mental health application or polythetic tools, given the availability of several relevant tools. While both are viable solutions, the concerns of the technological intervention are in the role played by the clinicians in the use of such tools, legal responsibility for the usage of technology tools, and keeping pace with the rapidly changing technology. The value of such tools, e.g., mindLAMP, lies in its facilitation of global collaboration [2]. It is interesting that globally popular features of the mindLAMP, as seen in the LAMP consortium of 54 global sites, are the surveys, data viewer, and location data collection. For patients with schizophrenia, the application engagement is a caregiving mechanism, complementary to the therapy provided by a clinician. A simple application interface improves the adoption of such technology, which is built into the design of the front end, and the back end can integrate complex capabilities, such as the interoperability of the LAMP platform with standards [3].

**Section S1.2. Comparative Visualization Design Pattern**

Dashboards have been actively used for decision-making in the healthcare sector, especially by healthcare workers, and such usage of technology is critical for decision-making [4]. Dashboards are essential for creating composite visualization views. Individual visualizations are effective in making sense of the data in digital psychiatry and in building data science workflows using artificial intelligence (AI) [5, 6]. Composing several visualizations together on a dashboard augments the understanding of the data.

There are specific design paradigms that are used in dashboards, of which comparative visualization patterns are useful for multivariate data. Such dashboards can be extended to different stakeholders, e.g.,patients and ICU staff to perform decision-making or evaluate the performance of the emergency department of the hospital [4].

There are information visualization design patterns for comparative visualizations, which include juxtaposition, superimposition, explicit encoding of difference, and explicit encoding of time warp[7]. Of these, juxtaposition has the weakest or lazy coupling of visualizations in a composite view, thus having the benefit of the visualizations being independent and non-interfering [8]. Despite its popularity as a design pattern, the pure juxtaposition strategy poses a challenge where it is the onus of the viewer to identify the connection between the visualizations [7]. Here, we address this challenge by allowing either the visualizations of different data slices of the same patient, or the same visualizations of different patients. Thus, we ensure a natural connection between the two visualizations in our proposed comparative visualization dashboard.

**Section S1.3. Visualization Methods**

In mindLAMPVis, we use several commonly used data analyses, such as multiple correspondence analysis (MCA), and appropriate visualizations of the outcomes. As an uncommon usage of clock glyphs [9, 10], we use them to represent the time spent in different significant locations [11]. Clock glyphs are effective for identifying temporal locations, especially owing to their radial encoding. We have used clock glyphs akin to pie chart glyphs [12, 13] to indicate the total time segments spent by the concerned patients in different locations (e.g.,home, work, etc.) during an entire day. These glyphs are then used to represent each date on a calendar view, unlike a packed circle layout used in ClockMap [14]. The use of the clock metaphor for a 24-hour day is effective for visualizing daily data, especially of patients [15]. Our proposed methods complement the anomaly plots generated from mindLAMP [16].

**Section S1.4. Design Study Methodology**

Visualization projects tend to straddle between problem-driven and technique-driven design solutions for real-world problems [17]. Through the creation lifecycle of mindLAMPVis, its design transitioned from the problem-driven to technique-driven status at the tail-end. Given it is predominantly problem-driven, we adopted the design study methodology (DSM) that is widely prescribed for visualization projects [17]. DSM involves a nine-stage framework which prominently features the collaboration or co-design of domain experts and visualization researchers. The sequential stages are are further subdivided to three top-level phases. By definition, the design study entails analysis, real-world problem, design, validation, and reflection. Thus, the phases and their constituent stages are sequential in nature, with feedback loops going from any stage to any of its preceding stages. These feedback loops make DSM highly iterative in theory, but in practice, the implementation allows only a few loops owing to the cost implication of each loop. Each phase includes some form of validation to indicate the completion of the phase.

Brief descriptions of the phases and stages of DSM framework are as follows

1. *Precondition Phase*: These are the steps towards the decision of undertaking the project and hence, mark the beginning of the design study. The validation of this phase is personal, i.e., decisions made by the collaborators.
   1. *Learn-Stage*: This stage involves understanding the state of the art designs through a systematic review of visualization literature.
   2. *Winnow-Stage*: This stage involves establishing a collaboration with practical, intellectual, and inter-personal considerations. For example, this is the stage to check accessibility to real data, time allocated for the project, extent of the problem to solve, rapport between team members, etc.
   3. *Cast-Stage*: This stage involves casting the roles of all team members involved in the project based on their expertise and skills.
2. *Core Phase*: These are the steps towards conducting the actual design study and a pilot implementation. The validation of this phase is inward-facing, i.e., the team makes course corrections as required.
   1. *Discover-Stage*: The actual problem characteristizatio along with its abstraction is completed at this stage.
   2. *Design-Stage*: The data representation, choice of visualization methods, and user interaction design are completed in this stage. The choice of specifications of system design and implementation, e.g., application libraries, is decided during this stage.
   3. *Implement-Stage*: This stage involves the software development of prototypes and tools, and usability engineering at feature level.
   4. *Deploy-Stage*: This stage involves release within the team and gathering feedback about the tool and its usability.
3. *Analysis Phase*: These are the steps towards reflecting the learnings from the design study and its implementation. The validation of this phase is outward-facing, i.e., the larger user-base and the outside world are involved in validating the final output.
   1. *Reflect-Stage*: This stage involves confirming, refining, and rejecting design ideas at large, and proposing new design guidelines for similar problem statements.
   2. *Write-Stage*: This stage involves writing a design study paper with a goal of knowledge transfer and not reproducibility.

**Section S2. Materials and Methods - in Detail**

Our proposed dashboard, mindLAMPVis is a comparative visualization tool using juxtaposed views. We propose the use of direct and derived data on the dashboard as multivariate data. Appropriate visualizations are used for each slice of the data based on its type and purpose for comparison.

**Section S2.1. Data Source and Description**

The data recorded on the mindLAMP application can be described as big data in terms of four Vs, namely, volume, velocity, variety, and value. Hence, the data needs to be processed and sampled appropriately before it can be used on mindLAMPVis. The dataset is from a two-country, three-site longitudinal study conducted as the Smartphone Health Assessment for Relapse Prevention (SHARP) project [16]. Of the total 132 study participants, 76 had a schizophrenia diagnosis. Of these 20 participants had clinical relapses before August 1st, 2022. The longitudinal study was conducted for a mean of 120+ days in the three sites. For our pilot study of mindLAMPVis, we have used the data from 24 patients out of the 76, of which a few of them had schizophrenic relapse events.

The data collected by the application is classified based on the mode of data collection:

- **Active data**: Data collected by the application while an individual is actively interacting with the application is referred to as active data. Surveys, cognitive tests, etc., constitute this form of data collection. Six surveys along with notifications for two were used as active data in the SHARP study [16]. Active data quality is determined using the ratio of completed to the expected surveys by each patient.
- **Passive data**: Data collected with the patient’s consent by the mindLAMP application in the background, without any voluntary activity by the patient, is referred to as passive data. GPS information, text, and call logs constitute this form of data collection. Thus, data streams from the different sensors, namely, accelerometer, GPS, and screen state, have been used as passive data in the SHARP study.

Since the data was collected in active and passive forms through multiple modalities across several patients in multiple cohorts, the dataset is rich, thus presenting several opportunities for data analysis. Here, we focus on some of the understudied slices of the data, namely, the survey data (active data) and the significant locations (passive data) [3]. The latter is the metrics of the GPS data [18], which is processed, condensed, and derived product of the raw data. The choice of the focus on survey data and significant locations was also based on the lower rate of missing values for the cohorts in the current study. Thus, the data preloaded in our visualization dashboard is of these variables. Characteristics of these variables are as follows:

- **Survey data**: The survey data was collected from individuals through a questionnaire. Most of the questions in the survey have Likert scale responses. Hence, the survey data, in computer science parlance, is nominal categorical data collected from each patient. The questions in the survey are broadly divided into five categories that are used as markers for the mental state of the patient. These categories are mood, anxiety, social, sleep, and psychosis.
- **Significant locations**: The mindLAMP application collects raw GPS data from individuals. These GPS coordinates are then clustered within the application to estimate the significant location data for each patient. The data resulting from clustering is a day-wise list of significant location coordinates, with the proportion of the day spent at that location and an estimated radius of the significant location.

**Section S2.2. Implementation of Design Study Methodology**

For the pilot implementation of mindLAMPVis, we have completed all the phases of DSM, except that the analysis- and reflect-stages are executed partially. In the cast-stage in our work, we determined that the clinicians play the role of both designers and end-users. The mindLAMP developers provided support of the existing software and access to data, and the visualization tool developers determined the data processing algorithms, visualization methods, and the actual implementation. The entire team participated in the deploy- and write-stages.

**Section S2.3. Imputation of Missing Data**

To build meaningful visualizations, it is necessary to handle missing data, which is inherent to digital phenotyping. Since the significant locations are derived data, we render the missing data as-is in its visualization. However, for the survey data, it is common practice to impute the data [19]. We impute the data using the following methods:

1. Last Observation Carried Forward (LOCF)
2. Multiple Imputation by Chained Equations (MICE)

LOCF method is a simple method for a pilot study, even though it is not suitable for some instances of medical applications [20]. MICE has been proven to be effective in such cases [20], which prompted us to test the same for our application. LOCF replaces missing values with the last available observation within the collection wave and hence is a basic method without high computational cost. While LOCF is the third most used imputation method, multiple imputation (MI) methods are considered more efficient. MI uses different simulation models to provide multiple values to the missing data instance. MICE assumes that a multivariate distribution can be used for the incomplete variable with missing data [21]. Hence, it uses multiple initial conditional distributions for the variable, and then MICE constructs a Gibbs sampling from the conditionals. The sampler is used for computing multiple imputations. MICE imputation is effective for resolving missing at random (MAR) and missing completely at random (MCAR) types [20].

**Section S2.4. Dimensionality Reduction**

The significant location data derived from the mindLAMP application is processed and can be directly visualized. For survey data, there are multiple sections with several questions in each. The *survey sections* included in our study are on mood, sleep, anxiety, social, and psychosis factors [18]. To visualize the data, this high-dimensional survey data needs to be transformed into low-dimensional data.

Given the categorical data format of survey responses, we use Multiple Correspondence Analysis (MCA) for dimensionality reduction. MCA is equivalent to Principal Component Analysis (PCA) but is for nominal categorical variables and was first proposed by Pierre Bourdieu [22]. MCA implementation includes outlier removal and determination of statistically significant relationships between categorical data points.

For each patient and each survey section, we use the time series of the survey responses as a dataset for dimensionality reduction. After discarding the timestamp information of the responses, each data point in the dataset, we treat the questions in each survey section as its dimensionality. We use MCA on these high-dimensional points to give a low-dimensional representation of the responses of the patient to the specific survey section. After performing dimensionality reduction, we add the timestamps back to the lower-dimensional data points, thus generating a time series of the transformed responses. It is important to note that we cluster the data points for each patient separately to preserve his/her/their unique behavioral patterns. This is to ensure that the trends are observed at an individual level through this visualization of MCA outcomes as opposed to the population level. To study the trend and correlation, we use the projection of each data point in the low-dimensional space defined by the MCA components.

We also compute the eigen-gaps in MCA as processed outcomes. Like PCA, MCA is computed by the eigenvalue decomposition of the covariance matrix of the data points. Here, we take the eigenvalues along the MCA components, which are the eigenvectors. The ith eigen-gap implies the significance of the ith MCA component relative to that of the (i + 1)th MCA. Thus, the first eigen-gap is the ratio of the first and the second eigenvalues for each survey section, and the second eigen-gap is the ratio of the second and third eigenvalues. These measures help us to determine how many MCA components of the data are required to describe the survey responses. Fewer significant (non-zero) MCA eigenvalues say one or two, indicate that the responses to the questions in the survey section are highly consistent during the time period.

**Section S2.5. Visualizations**

We integrate the following visualizations of active (survey data) and passive data (significant locations/GPS data) in the mindLAMPVis dashboard:

1. MCA Trend (V1A) of survey data
2. MCA Eigen-gap (V2A) of survey data
3. Date-Clustering (V3A) of survey data
4. Hometime (V1P) of significant locations data
5. Significant Location (V2P) of significant locations data

Each of these visualizations represents a specific *feature* of the patient data. Each feature is further uniquely described by its *parameters*. The goal of mindLAMPVis is to provide interactivity through user-defined choices of patients, features, and feature parameters.

In this section, we describe various methods using which we visualize a patient’s data to derive insights into the patient’s recovery, relapse, and subsequent treatment plans.

- **Survey MCA Trend (V1A)**: We consider the survey MCA time series to be a feature here. To implement the MCA, we need to choose the imputation methods (LOCF and MICE) and the survey sections, i.e., mood, sleep, anxiety, social, and psychosis factors. Thus, the implementation method and survey section are treated as feature parameters.

The chosen visualization for this feature is a time series plot of the projection of data onto the first MCA component of the selected feature parameter (Figure 1).

Higher Y-values imply a high variation in the responses for the questions of the survey category. This can be interpreted as being proportional to the degree of truthfulness in the responses, i.e.,the effort put in by the patient to respond to the questions.

- **Survey MCA Eigen-Gap (V2A)**: As discussed in Section 2.4, eigen-gap helps us understand how many dimensions of the MCA components of the survey data are important for each patient. For instance, a high first eigen-gap implies that the second MCA component can be safely ignored. In this scenario, one can use the V1A visualization that makes use of the first MCA components exclusively and derives insights. In the eigen-gap visualization, our goal is to compare the values across different survey sections for each patient. The feature here is the vector of the first three MCA eigenvalues and the feature parameters are the ranking of the eigen-gap, i.e., first or second eigen-gap, and the imputation method for MCA.

The eigen-gap is visualized using overlapping bar charts (Figure 2). Overlapping bar charts are helpful when different categories of the data follow a known inequality, i.e.,the ith MCA eigenvalue is always greater than (i + 1)th one. The larger MCA eigenvalue is represented using a thicker bar at the back, and the smaller one is superimposed as the thinner bar. Each set of thick and thin bars corresponds to the section of the survey questionnaire. Overall, visualizing the eigen-gap provides insight into the variance available in the responses in each category over time for the concerned patient.

An alternative visualization is a simple bar chart depicting the direct encoding of the eigen-gap as a ratio. However, when second and/or third MCA eigenvalues are zero, the eigen gap becomes NaN, leading to invalid visualizations. Hence, we discarded the alternative visualization design.

- **Survey Date-Clusters (V3A):** The feature analyzed here is the calendar date of the survey response for each patient. We cluster the calendar dates based on the responses to the survey questions. The rationale behind date-clusters is to determine temporally similar behavior of the patient, intending to find recurring patterns.

We choose to use *unsupervised clustering* techniques in the absence of ground truth of date-clusters. For implementing such clustering methods, we represent each date of a patient’s survey response as a feature vector. The feature vector is determined based on the aggregation of the survey questions, for which we propose two strategies, namely, aggregated and complete vectors. Let us define N_S_ to be the number of questions in the

survey. Suppose the survey has M sections, where the ith section has N_S_^(i)^ questions, such that ∑^M^_i=1_ N_S_^(i)^ = N_S_.

**Aggregate Vector (AV)**: An aggregate vector is one composed of the average value of the responses for each survey section for a calendar date of a patient. Thus, this vector is of size M. We ensure that all responses are semantically the same, which implies they are all increasing or are all decreasing.

**Complete Vector (CV)**: A complete vector is one where the responses to all questions are considered individually considered is computed for a calendar date of a patient. Thus, this vector is of size N_S_.

For clustering the vectors representing calendar dates for a patient, we use two different algorithms:

**Natural Clustering (NClust)**: Here, we use algorithms that do not require a predefined cluster count. The algorithms used here are affinity propagation, DBSCAN, Mean shift, and OPTICS.

**Predefined Clustering (KClust)**: Here, we specify the number of clusters *k* which the user can select on mindLAMPVis. Our tool allows the user to select k from the following values: 2, 3, 5, 10, 15. The algorithms used for a selected *k*-value are BIRCH, Agglomerated Clustering, K-means, Mini-batch K-means, Spectral Clustering, and Gaussian Mixture Models.

**Consensus Voting of Co-association Matrices**: In the absence of ground truth of the clusters, we perform consensus clustering [22]. We take m clustering algorithms for a selected clustering requirement, e.g., selected patient. For each clustering algorithm, we compute a **co-association matrix** (C) of size N, which is the number of calendar dates of surveys available for the patient.

**Definition 2.1 (Co-association Matrix)**. In the N × N matrix, an element **C_k_**(i, j) is one if the ith and the jth calendar dates belong to the same cluster in the kth clustering algorithm; and zero, otherwise. Thus, these matrices are binary. Then, we average the m co-association matrices to compute the consensus of co-association matrices Cµ . Each matrix element **C_c_**(i, j) gives the likelihood of the ith and jth calendar dates being in the same cluster. Thus, the elements of **C_µ_** are real values between 0 and 1. We visualize **C_µ_** using matrix visualization where the likelihood value from 0 to 1 is colored using a gradient color palette (Figure 3).

To improve the readability of the matrix visualizations, we use seriation algorithms to rearrange the rows and columns so that the ordering of the calendar dates along the rows and columns is the same [24]. Seriation leads to observations of block-like structures along the diagonal. *These block-like structures indicate a strong tendency of clustering of the rows (or, equivalently the columns) within each of the blocks.*

We use eight different seriation algorithms: by date, by frequency, by cluster, by optimal leaf ordering, by leaf ordering over distance matrix, by cross-width reduction, by bandwidth reduction, and by spectral ordering. Except for the first three methods of directly sorting the dates, the remaining algorithms are adopted from Reorder.js [25].

For the algorithms that require a distance matrix **D,** options are provided for the computation of the distance matrix using different distance metrics, namely, Manhattan, Euclidean, Chebyshev, Hamming, Jaccard, and Bray-Curtis.

For most of the patient data, we observed that the *optimal leaf ordering* seriation generates matrix visualizations with clear clusters in the diagonal for our application. Optimal leaf ordering, as used for matrix seriation [25], internally uses hierarchical clustering originally proposed for gene-expression data analysis. This iterative algorithm is an agglomerative clustering one. It uses a distance matrix **D**, starting with all data points as leaves of a tree and as singleton clusters, and combines clusters with the least inter-cluster distance in each iteration. The inter-cluster distance is computed using the maximum linkage clustering criterion, i.e., the maximum distance between data points across the clusters.

- **Hometime (V1P)**: Hometime gives the value of the amount of time spent by the patient at home. In mindLAMP, this feature is given as a ratio of the hometime to the entire day. To distinguish between days with unavailable data and those with zero hometime data, we use color encoding. The former is colored using gray and the latter using a gradient color palette.

To arrive at a color for days with available data, we determine the minimum percentage of hometime for the month for the patient within the available data, hmin . We then map the hometime ratio to gradient color map between h_min_ and 1.0. This helps in visualizing the variations of hometime across weeks in the month more effectively. Thus, the feature is the hometime ratio and the feature parameter is the choice of a calendar month within the available data. Below the choice widget for the calendar month, the information on the range of months of available data is provided as text.

We visualize the hometime data as square glyphs in the calendar visualization (Figure 4). The color of the glyphs provides inferences of anomaly in hometime trends. The highest and least times spent at home, in that month, are at the extremes of this color palette. This helps in identifying anomalies in the patient’s behavior around the relapse time.

- **Significant Locations (V2P):** The feature corresponding to the significant locations is a vector of significant locations visited during a calendar day along with the percentage of time spent by the patient at each of them during the day. The feature parameter is the choice of month, similar to V1P.

Since this is compositional data, we use pie chart glyphs in the calendar view for a month (Figure 4), similar to the visualization of V1P. The color coding uses a categorical color palette to distinguish between different locations within each pie chart.

This visualization helps us to identify month-wise anomalies in the proportion of time spent at significant locations each day. The highly fragmented pattern of the pie glyph indicates the high mobility of the patient during the day. We compare the pie chart glyphs before and after relapse to observe relevant behavioral patterns in the mobility of the patient.

**Additional Visualization – Survey MCA Correlation**: We consider the vector of the first two MCA components as the feature. As implemented in V1A, we use projections for the first two MCA components in the visualization of the feature vector. Since this involves the implementation of MCA, we use the same feature parameters as in V1A.

The chosen visualization for this feature is a scatterplot of the data projections onto the first and second MCA components (Figure 5), represented along the *X-* and *Y -*axes, respectively. The color encoding of the scatter points is based on the calendar date corresponding to the data item. A gradient color palette is used to represent the range of time from the oldest to the newest calendar date in the data available for the selected patient.

The points closer to the X-axis indicate that those data points are closer to the corresponding first MCA component than to the second component. Such points imply the variance in the responses for the data point can be represented by the first MCA component axis alone. Other points have a significant second MCA component value that must not be ignored upfront.


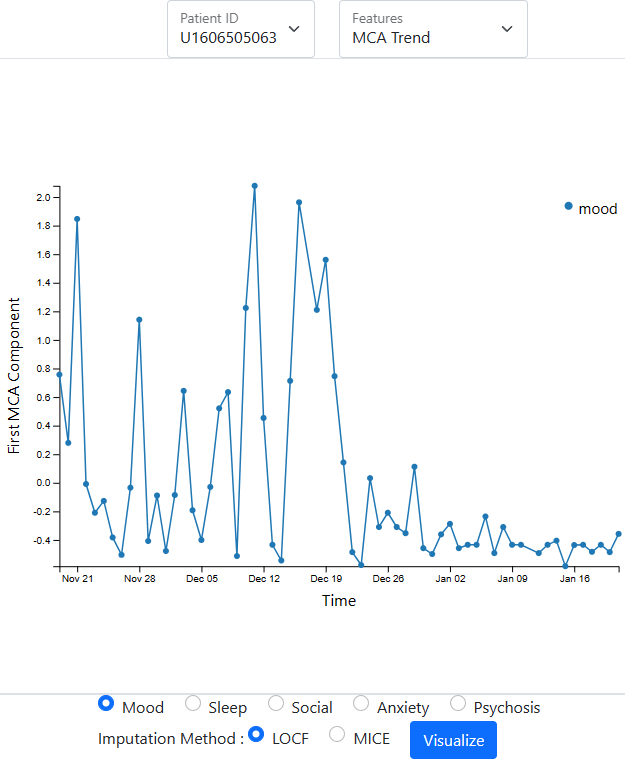


**Figure S1.** Time-series plot of data projection onto the first MCA component for selected survey section and imputation method, for a selected patient. This chart is generated from the anonymized data of a patient from the Bangalore cohort collected during 2021-2022 in the SHARP project [16].


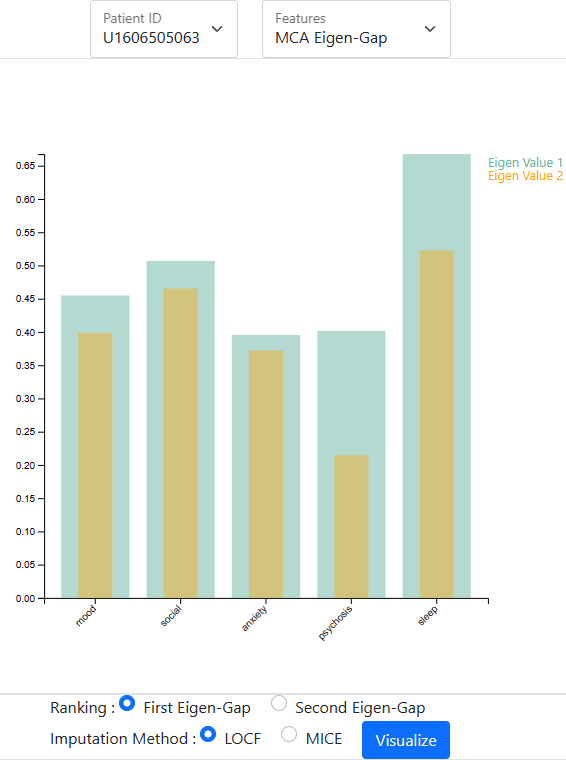


**Figure S2.** Overlapping bar chart visualization of the eigen-gap of the MCA of the survey by visualizing the concerned eigenvalues in each bar, for comparing the eigen-gaps across survey sections for the chosen ranking of the eigen-gap for a selected patient. This chart is generated from the anonymized data of a patient from the Bangalore cohort collected during 2021-2022 in the SHARP project [16].


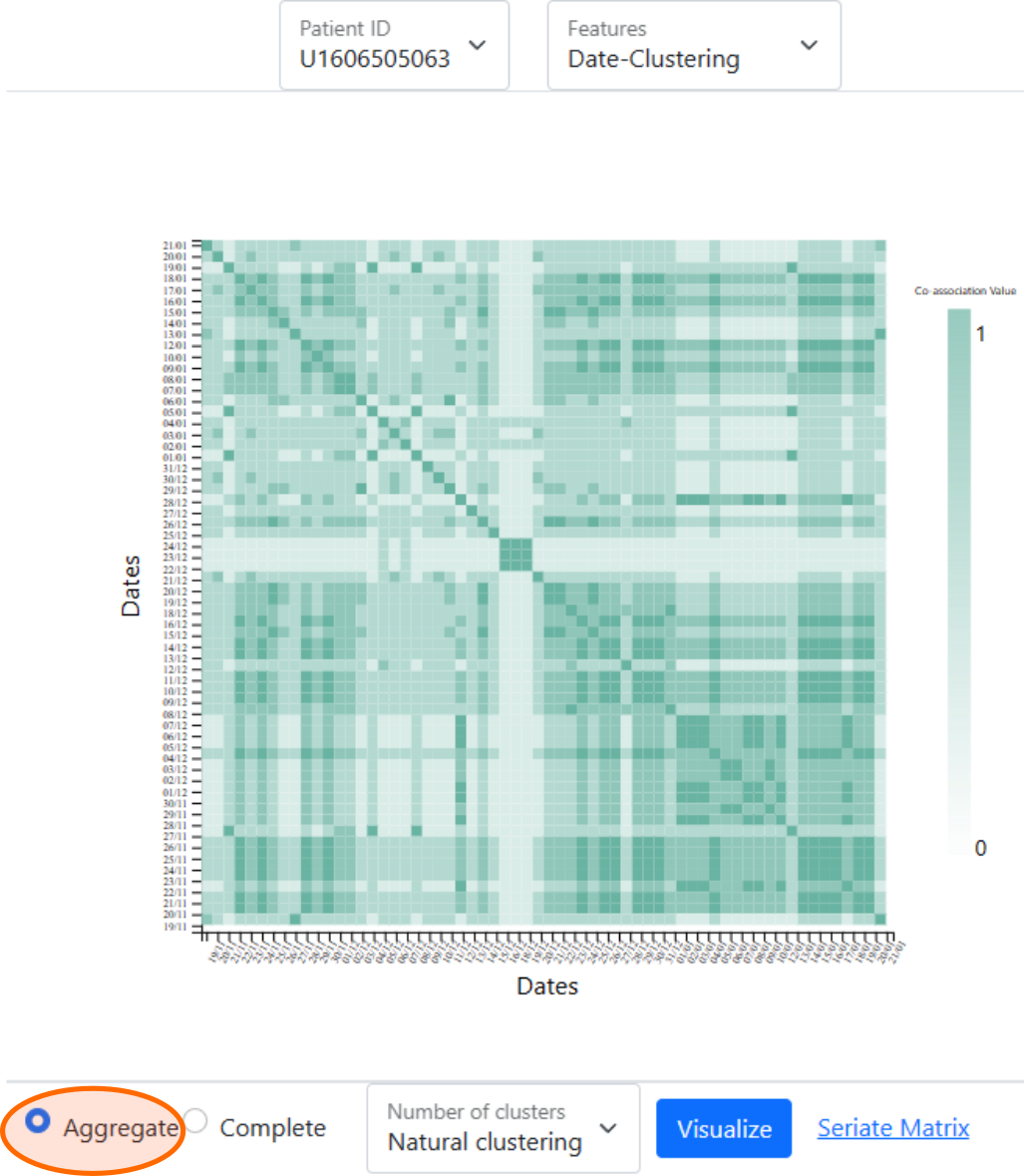


**Figure S3.** Matrix visualization of the consensus matrix of clustering of calendar dates for chosen aggregation and clustering strategies, for a selected patient. The orange highlight shows the choice of aggregate vector (AV) for this example. This chart is generated from the anonymized data of a patient from the Bangalore cohort collected during 2021-2022 in the SHARP project [16].

**
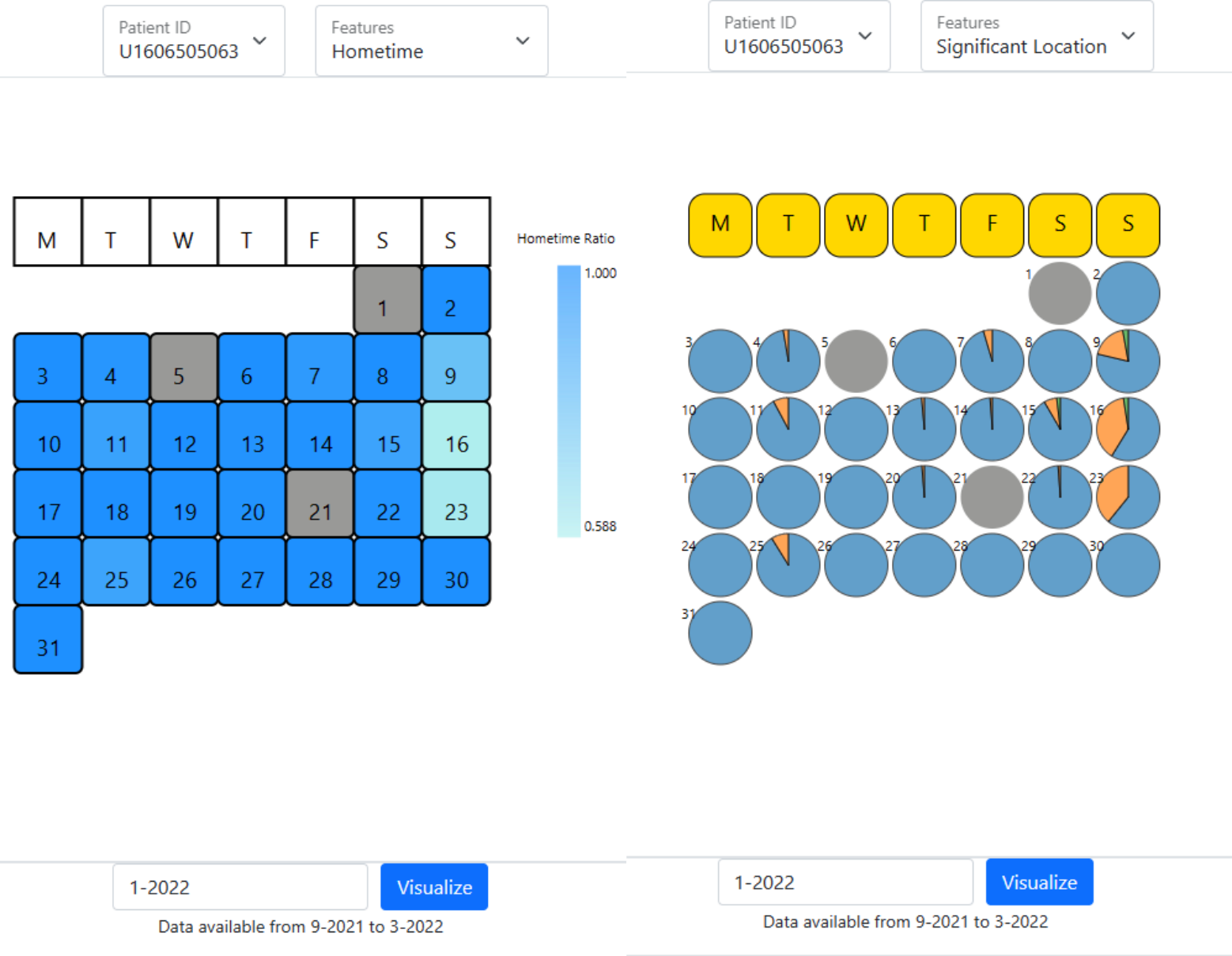
**

**Figure S4.** A patient-specific calendar view visualizations of (Left) hometime (V1P) showing the percentage of hometime colored using gradient color palette, (Right) the distribution of significant locations (V2P) using pie-chart glyphs. This chart is generated from the anonymized data of a patient from the Bangalore cohort collected during 2021-2022 in the SHARP project [16].


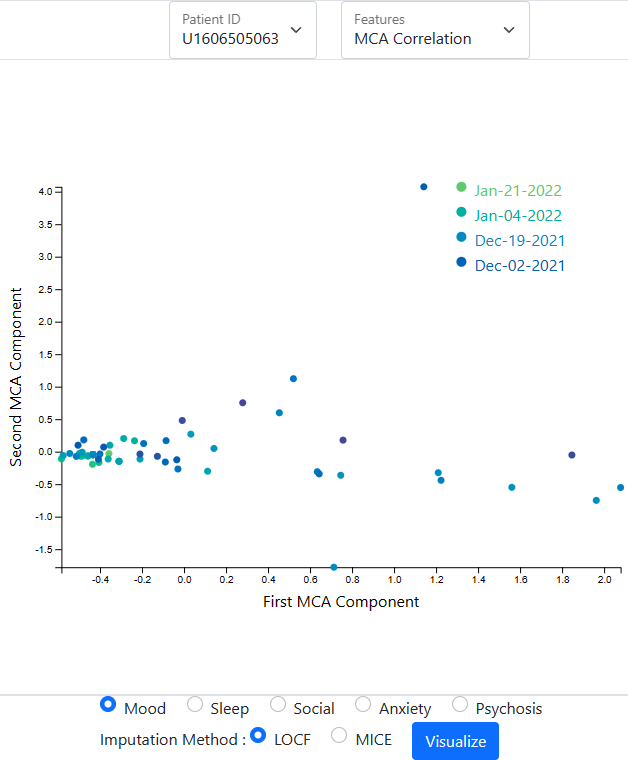


**Figure S5.** Scatterplot visualization of the data projections onto the first two MCA components for the selected survey section and imputation method, for a selected patient. This chart is generated from the anonymized data of a patient from the Bangalore cohort collected during 2021-2022 in the SHARP project [16].

**Section S3. mindLAMPVis GUI**

The graphical user interface (GUI) of mindLAMPVis consists of two equal vertical halves, referred to as *panels*, that are juxtaposed, for comparative visualization (Figure 6). Each panel consists of a canvas region for visualization display and corresponding widgets (dropdown menus, buttons, etc.) which helps the user to select the patient by ID and the visualization method, thus choosing the *feature* to be visualized.

Once the data is preloaded, the user can select the feature to be visualized using *feature-controls*. For every feature, there are parameters specific to the feature that need to be selected, for which we have the *feature-parameter-controls*. The *canvas* is the drawing region. The feature-control widgets for the choice of patient ID and feature are placed above the canvas, and the feature-parameter-control widgets are placed below the canvas (Figure 6).

When the application starts up, the canvases for both panels are blank. As the user updates the choices of patient ID, features, and feature parameters, the visualizations are generated in real-time, thus making the application interactive.

**Section S3.1. Implementation**

The software architecture for the implementation of mindLAMPVis is given in Figure 7.

**Workflow**: When the user interactively sets various options in the feature-controls and feature-parameter-controls, the change of options on the interface is submitted to the flask application at the server. Based on the options selected, relevant data is loaded into the mindLAMPVis application by reading the relevant pre-formatted user data files. The new data is then pushed to a JavaScript module, which triggers and maps the data to the selected visualization module. The visualization modules are built using D3.js [26].

**Database**: We receive the patient’s activity data from the mindLAMP database. We preprocess this data into different files, storing essential information as comma-separated value files. With the data stored in designated paths on the server – depending on the features selected by a user, the flask application reads the relevant files and loads data at runtime.

**Deployment**: We have hosted our app on Azure services and integrated the workflow with Github. Thus, when verified code changes are pushed to GitHub, the GitHub actions integrated with Azure auto-deploy the changes onto our live web application. Our visualization dashboard mindLAMPVis is available online [27]. The source code for the app with a dummy input dataset is also available online [28].

**Section S3.2. Comparison with Anomaly Plots**

Using both sensor and survey data as passive and active acquisitions, respectively, for relapse prediction in psychosis is highly feasible [29]. Anomaly detection is an effective data analysis method used to identify data points that significantly deviate from the majority, indicating potential errors, unusual events, or significant changes in the observed process. These algorithms enable researchers and clinicians to longitudinally identify abnormal deviations from an individual’s typical sensor and survey data, which is indicative of relapse [16]. The anomaly plot (shown in Figure 8) is the visual representation of potential relapse events and unusual data behavior, providing valuable insights for monitoring schizophrenia relapse.

**Interpreting Anomaly Plots**: The x-axis represents the time in days since the first data point, while the y-axis shows the anomaly detection p-value plotted on an inverse logarithmic scale. The plot has passive data (purple points). To smooth out noise in the passive data, a running minimum is calculated and plotted (black line). The yellow highlights show periods with sustained anomalous passive data measurements, indicating significant deviations from the expected pattern.

**
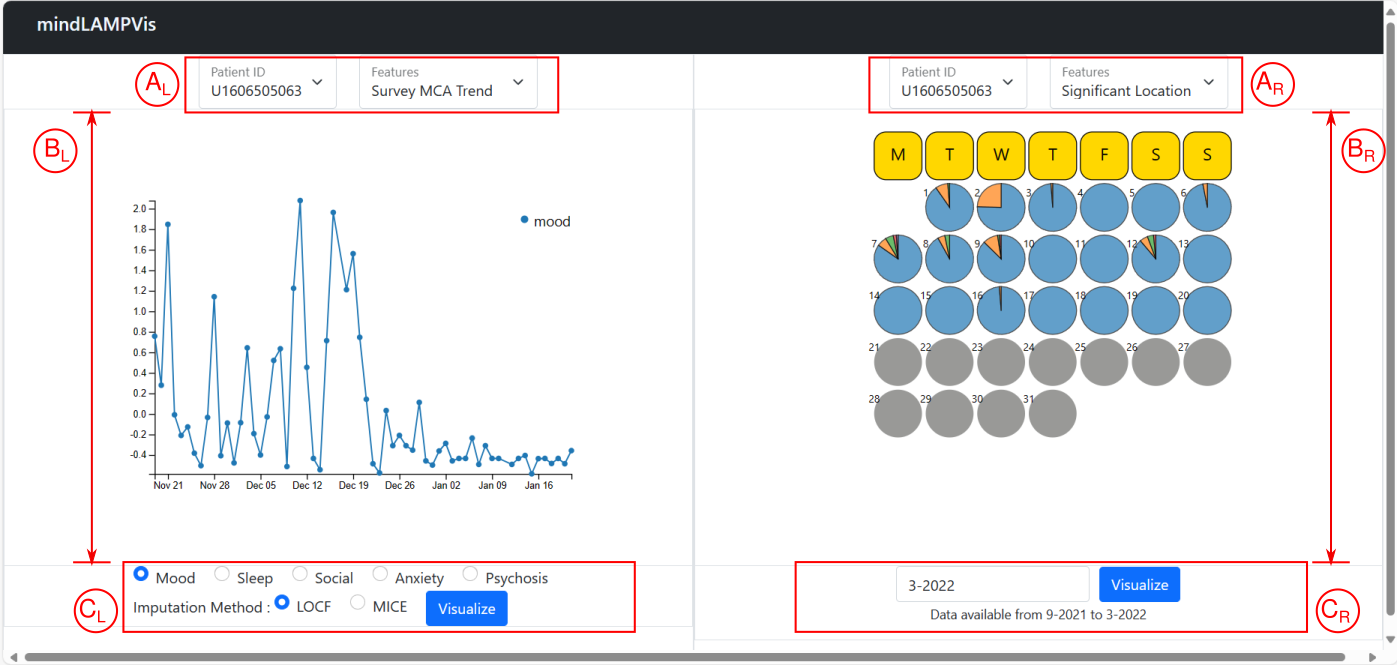
**

**Figure S6.** View of the two-panel application with annotations of the components of the GUI. A, B, and C indicate feature-controls, canvas, and feature-parameter-controls, respectively, and the subscripts L/R indicate the left/right panels, respectively. The left and right panels have the same design with *clean lines*. These charts in the screenshot are generated from the anonymized data of a patient from the Bangalore cohort collected during 2021-2022 in the SHARP project [16].


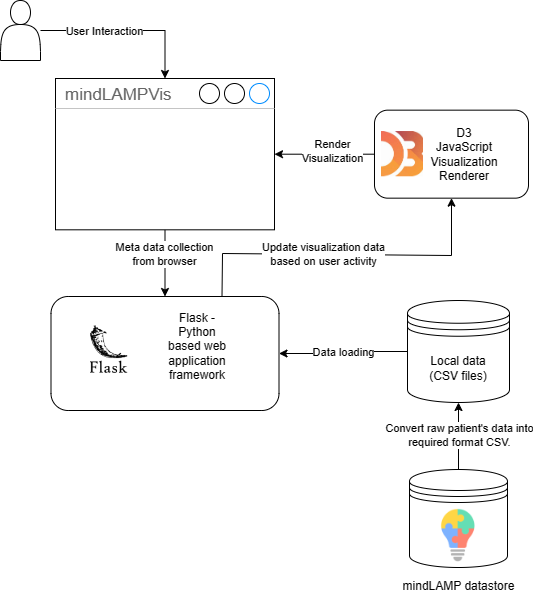


**Figure S7.** Software architecture of mindLAMPVis.

**
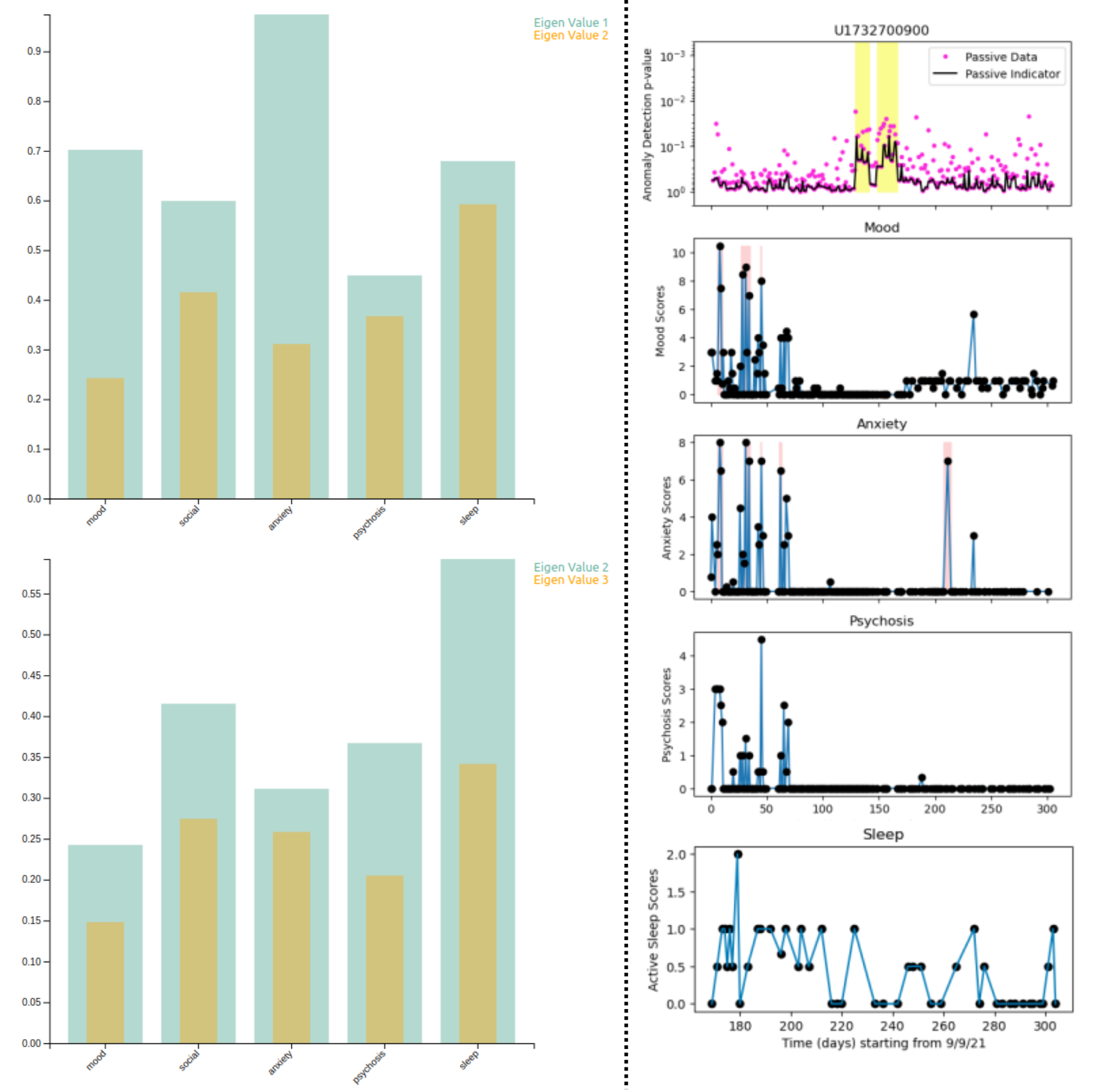
**

**Figure S8.** MCA eigen-gap (V2A) visualization for Patient A (discussed in detail as a case study in Section 3 of the main paper) using LOCF imputation for the five survey sections, shows significant first and second eigen-gaps in (Left) *Anxiety* and *Sleep*, respectively. We observe similar patterns in (Right) the anomaly plot for the same period. This chart is generated from the anonymized data of the patient from the Bangalore cohort collected during 2021-2022 in the SHARP project [16]. The clinicians logged a relapse in April 2022 for this patient.

**References**

[1] Robert E Drake. Mental health technology tools: Two alternative approaches. Epidemiology and Psychiatric Sciences, 29:e99, 2020.

[2] Rebecca Bilden and John Torous. Global collaboration around digital mental health: The LAMP consortium. Journal of Technology in Behavioral Science, 7(2):227–233, 2022.

[3] Aditya Vaidyam, John Halamka, John Torous, et al. Enabling research and clinical use of patient-generated health data (the mindLAMP Platform): digital phenotyping study. JMIR mHealth and uHealth, 10(1):e30557, 2022.

[4] Aleeha Iftikhar, Raymond Bond, Victoria McGilligan, Stephen J Leslie, Khaled Rjoob, Charles Knoery, and Aaron Peace. Role of dashboards in improving decision making in healthcare: Review of the literature. In Proceedings of the 31st European Conference on Cognitive Ergonomics, pages 215–219, 2019.

[5] Adwitiya Ray, Akansha Bhardwaj, Yogender Kumar Malik, Shipra Singh, and Rajiv Gupta. Artificial intelligence and Psychiatry: An overview. Asian Journal of Psychiatry, 70:103021, 2022.

[6] Wen Zhou, Xue Bai, Yun Yang, Miao Huang, Qiulan Zheng, Jiaqian Wu, Rui Wang, and Xiuni Gan. Revelations of delirium subtype research: A bibliometric analysis of publications in the past twenty years in the field. Asian Journal of Psychiatry, 83:103561, 2023.

[7] Michael Gleicher, Danielle Albers, Rick Walker, Ilir Jusufi, Charles D Hansen, and Jonathan C Roberts. Visual Comparison for Information Visualization. Information Visualization, 10(4):289–309, 2011.

[8] Waqas Javed and Niklas Elmqvist. Exploring the design space of composite visualization. In 2012 IEEE Pacific Visualization Symposium, pages 1–8. IEEE, 2012.

[9] Johannes Fuchs, Fabian Fischer, Florian Mansmann, Enrico Bertini, and Petra Isenberg. Evaluation of alternative glyph designs for time series data in a small multiple setting. In Proceedings of the SIGCHI Conference on Human Factors in Computing Systems, pages 3237–3246, 2013.

[10] Johannes Hermann Fuchs. Glyph design for temporal and multi-dimensional data: Design considerations and evaluation. PhD thesis, Universität Konstanz, Germany, 2015.

[11] Tanvi Lakhtakia, Ameya Bondre, Prabhat Kumar Chand, Nirmal Chaturvedi, Soumya Choudhary, Danielle Currey, Siddharth Dutt, Azaz Khan, Mohit Kumar, Snehil Gupta, et al. Smartphone digital phenotyping, surveys, and cognitive assessments for global mental health: Initial data and clinical correlations from an international first episode psychosis study. Digital Health, 8:20552076221133758, 2022.

[12] Jason Pearlman and Penny Rheingans. Visualizing network security events using compound glyphs from a service-oriented perspective. In VizSEC 2007: Proceedings of the Workshop on Visualization for Computer Security, pages 131–146. Springer, 2008.

[13] Artem Amirkhanov, Bernhard Fröhler, Johann Kastner, Eduard Gröller, and Christoph Heinzl. InSpectr: Multi-Modal Exploration, Visualization, and Analysis of Spectral Data. In Computer Graphics Forum, volume 33, pages 91–100. Wiley Online Library, 2014.

[14] Fabian Fischer, Johannes Fuchs, and Florian Mansmann. ClockMap: Enhancing Circular Treemaps with Temporal Glyphs for Time-Series Data. In EuroVis (Short Papers), 2012.

[15] Majedah Alrehiely. Evaluating different visualization designs for multivariate personal health data. PhD thesis, Cardiff University, 2020.

[16] Asher Cohen, John A Naslund, Sarah Chang, Srilakshmi Nagendra, Anant Bhan, Abhijit Rozatkar, Jagadisha Thirthalli, Ameya Bondre, Deepak Tugnawat, Preethi V Reddy, et al. Relapse prediction in schizophrenia with smartphone digital phenotyping during COVID-19: a prospective, three-site, two-country, longitudinal study. Schizophrenia, 9(1):6, 2023.

[17] Michael Sedlmair, Miriah Meyer, and Tamara Munzner. Design study methodology: Reflections from the trenches and the stacks. IEEE Transactions on Visualization and Computer Graphics, 18(12):2431–2440, 2012.

[18] Elena Rodriguez-Villa, Urvakhsh Meherwan Mehta, John Naslund, Deepak Tugnawat, Snehil Gupta, Jagadisha Thirtalli, Anant Bhan, Vikram Patel, Prabhat Kumar Chand, Abhijit Rozatkar, et al. Smartphone Health Assessment for Relapse Prevention (SHARP): a digital solution toward global mental health. BJPsych Open, 7(1):e29, 2021.

[19] Sarah Chang, Noy Alon, and John Torous. An exploratory analysis of the effect size of the mobile mental health Application, mindLAMP. Digital Health, 9:20552076231187244, 2023.

[20] Matthijs Blankers, Maarten WJ Koeter, Gerard M Schippers, et al. Missing data approaches in eHealth research: Simulation study and a tutorial for nonmathematically inclined researchers. Journal of Medical Internet Research, 12(5):e1448, 2010.

[21] Stef Van Buuren and Karin Oudshoorn. Flexible multivariate imputation by MICE. Leiden: TNO, 1999.

[22] Frédéric Lebaron. How Bourdieu "quantified" Bourdieu: The geometric modelling of data. In Quantifying Theory: Pierre Bourdieu, pages 11–29. Springer, 2009.

[23] Reddy Rani Vangimalla and Jaya Sreevalsan-Nair. A multiscale consensus method using factor analysis to extract modular regions in the functional brain network. In 2020 42nd Annual International Conference of the IEEE Engineering in Medicine & Biology Society (EMBC), pages 2824–2828. IEEE, 2020.

[24] Innar Liiv. Seriation and matrix reordering methods: A historical overview. Statistical Analysis and Data Mining: The ASA Data Science Journal, 3(2):70–91, 2010.

[25] Jean-Daniel Fekete. Reorder.js: A javascript library to reorder tables and networks. In IEEE VIS 2015: Proceedings of the IEEE Visualization Conference, 2015.

[26] Michael Bostock, Vadim Ogievetsky, and Jeffrey Heer. D3 data-driven documents. IEEE Transactions on Visualization and Computer Graphics, 17(12):2301–2309, 2011.

[27] Karthik Sama, Jaya Sreevalsan-Nair, Soumya Choudhary, Srilakshmi Nagendra, Preethi V Reddy, Asher Cohen, Urvakhsh Meherwan Mehta, and John Torous. mindLAMPVis: Data Visualization Portal. <https://mindlampvis.azurewebsites.net/>, 2024.

[28] Karthik Sama, Jaya Sreevalsan-Nair, Soumya Choudhary, Srilakshmi Nagendra, Preethi V Reddy, Asher Cohen, Urvakhsh Meherwan Mehta, and John Torous. MindLAMPVisDev. <https://github.com/GVCL/mindLAMPVisDev/>, 2025.

[29] Philip Henson, Ryan D’Mello, Aditya Vaidyam, Matcheri Keshavan, and John Torous. Anomaly detection to predict relapse risk in schizophrenia. Translational Psychiatry, 11(1):28, 2021.
