# Supplementary material for: mindLAMPVis as a Co-Designed Clinician-Facing Data Visualization Portal to Integrate Clinical Observations From Digital Phenotyping in Schizophrenia: User-Centered Design Process and Pilot Implementation
Source: JMIR Form Res. 2025 Jun 10;9:e70073. doi: 10.2196/70073 (PMC12173093; doi:10.2196/70073)
Supplement: Checklist 1 [file formative-v9-e70073-s002.docx]

This multimedia appendix provides a checklist of adherence to the iCHECK-DH guidelines [1] for reporting the design study of mindLAMPVis. The seven sections with 20 items prescribed in the checklist are given as sections with headings, respectively. The items that are mandatory and non-mandatory to be reported are marked as “(M)” and “(NM),” respectively, in the headings within each section.

**Section S1. Title**

This section includes the mandatory item related to the title.

***Item 01: Title (M) –*** The title has terms “Data Visualization Portal” and “User-centered Design Process and Pilot Implementation” that sufficiently describe that the manuscript is an implementation report. The title includes the name of the app that is developed (mindLAMPVis), its unique design process (co-designed), its target audience (Clinician-facing), and its characteristic application (digital phenotyping in schizophrenia. Hence, the title is concise.

**Section S2. Abstract**

This section includes the mandatory item related to the “Abstract” of the manuscript.

***Item 02: Abstract (M) –*** The Abstract is structured as “background, objectives, methods, results, and discussion.” The methods discuss the use of multivariate analysis and dimensionality reduction for creating the visualizations. The key characteristics of the visualization portal have been mentioned. The key results from case studies conducted by the clinicians have been highlighted. All keywords have been discussed in the abstract and are listed at the end of the abstract.

**Section S3. Introduction**

This section includes the mandatory items related to the “Introduction” section of the manuscript.

***Item 03: Context (M) –*** The Introduction provides the context of the app with Bangalore as the target geographical area, patients with a schizophrenia diagnosis as the target population, and the pilot implementation. The following text in the Introduction sums it up:

*In this study, we propose mindLAMPVis, which is an interactive visualization tool designed to enhance the usability of data processed through the mindLAMP platform. The data is prepared for visualization using multivariate data mining through dimensionality reduction. The inspiration for the tool derives from the request and needs of local clinicians in Bangalore, India, after initial work with the mindLAMP platform for a research study.*

*...*

*We hypothesize that a comparative visualization tool improves the understanding and management of schizophrenia, thereby supporting more effective, timely interventions and fostering a better provider-patient relationship.*

The current stage of implementation is stated as:

*The pilot implementation of mindLAMPVis is available online [2]. The source code for the app with a dummy input dataset is also available online [3].*

***Item 04: Problem Statement (M)*** – The problem statement has been adequately described in the Introduction, as provided below:

*Despite the well-known potential of digital mental health to advance psychiatric care, a lack of clinical integration has hampered meaningful use [4]. Low and middle-income countries are ideal settings for these technologies to help advance care and reduce access barriers. However, many of the digital tools like apps need not be designed to cater to the clinicians’ needs [5] or locally developed [6], which affects the adoption of the technology.*

*...*

*Despite these benefits concerning patient data in general, the unique nature of digital phenotyping data has led to limited efforts in its visualizations. Previous works show the role of visualizations in aiding clinicians to understand patients’ health-related behavior from digital phenotyping data [7, 8]. However, such studies are limited to the analysis of passive data of patient behavior (such as physical location and smartphone usage patterns) by healthcare providers [9, 10]. Such studies also do not investigate the integrability of such analysis to the data management framework.*

**Item 05: Similar Interventions (M) –** Our work is designed to be integrated with the open-source digital phenotyping smartphone app, mindLAMP [11, 12]. The following is provided in the Introduction:

*This article explores one example of such tailoring by focusing on the use of the open-source mindLAMP smartphone app [11, 12], where clinicians and computer scientists co-design the visualization portal that is integrable with the mindLAMP app.*

**Section S4. Methods**

This section includes the mandatory items related to the “Materials and Methods” section of the manuscript.

***Item 06: Aim and Objectives (M) –*** The aim and objectives of our work are articulated in the Methods section as follows:

*Our proposed clinician-facing dashboard, mindLAMPVis, is intended to compare different visualizations of the same patient. For exploratory analysis, the same visualization of different patients can be compared. Our goal was to co-design the app with clinicians, visualization experts, and software developers in Bangalore and Boston to suit the local clinical needs in LMIC.*

***Item 07: Blueprint Summary (M) –*** The collaborative design process is described in detail in the Methods section under the heading “Co-design Process” which is derived from the design study methodology (DSM) [13]. DSM is widely used by the visualization research community for visualization project implementation in real-world scenarios.

***Item 08: Technical Design (M) –*** The computational methods are described briefly under the headings, “Imputation,” “Dimensionality Reduction,” “Clustering and Co-association Matrix,” and “Visualizations” in the Methods section. This section also describes the layout of the Graphical User Interface (GUI) of the mindLAMPVis app. The manuscript contains sufficient description of these methods to provide the JMIR Formative Research readership information on the technical design of mindLAMPVis.

A detailed description of these methods with mathematical rigor is given in the Multimedia Appendix 1. A section “mindLAMPVis GUI” in the Multimedia Appendix 1 gives a detailed description of the GUI design, the implementation using JavaScript and the mindLAMP database, and the deployment using Azure services and Github. The overview of the software architecture is

also provided in the Multimedia Appendix 1. The Methods section in the manuscript gives only sufficient technical design details to provide the narrative of the app design, implementation, and usage for the case study.

***Item 09: Target (M) –*** The population data that is targeted is that of the patients with a schizophrenia diagnosis. This is described under headings “Ethical Considerations” and “Clinical Setting ” in the Methods section of the manuscript.

The targeted audience of the mindLAMPVis app is the clinicians, who have been involved in the co-design of this app, as described in Item 07 above. There are no exclusive eligibility criteria for these clinicians, apart from being consumers of the digital phenotyping data from the mindLAMP app.

***Item 10: Data (M) –*** The data used for mindLAMPVis is the anonymized data from active and passive modalities of patients with schizophrenia, who were recruited for a multi-site, longitudinal observational study in the SHARP project [12]. The relevant details are provided in the Methods section under the heading “Dataset.” Further supporting details are available in the Multimedia Appendix I in the subsection “Data Source and Description.”

***Item 11: Interoperability (M) –*** We have not currently used any specific interfaces or standards, as the app is developed as a pilot implementation on the data collected using the mindLAMP app in the SHARP project [12]. We have retained the file formats followed in the mindLAMP app, for which dummy data is provided in the codebase published in the GitHub repository of mindLAMPVis [3].

***Item 12: Participating Entities (M) –*** The participating entities are hospitals, namely, the National Institute of Mental Health and Neuro Sciences (NIMHANS) in Bangalore and the Beth Israel Deaconess Medical Center (BIDMC) in Boston, and a technical university, namely, the International Institute of Information Technology, Bangalore (IIIT-B).

The roles of the participating entities are mentioned in the Methods section as follows:

*Co-design Process: The dashboard is an outcome of the co-design process, i.e., a collaborative design process, involving clinicians in Bangalore and Boston, mindLAMP developers in Boston, and computer scientists with visualization expertise from Bangalore.*

*...*

*For the pilot implementation of mindLAMPVis, we have completed all the phases, except that the analysis- and reflect-stages are executed only partially. In the cast-stage of our work, we determined that the clinicians play the role of both designers and end-users. The mindLAMP developers provided support for the existing software and access to data, and the visualization tool developers determined the data processing algorithms, visualization methods, and the actual implementation. The entire team participated in the deploy- and write-stages.*

The mindLAMPVis app has been developed as an academic project without any direct funds, whereas the mindLAMP app was developed with financial support from the Wellcome Trust.

***Item 13: Budget Planning (M) –*** This specific project, in its current implementation, did not involve any budget planning.

***Item 14: Sustainability (M) –*** This specific project will seek funds from the Government of India’s healthcare schemes to sustain further development and deployment at scale.

**Section S5. Implementation**

This section includes the mandatory items related to the “Results from Case Studies” section of the manuscript.

***Item 15: Coverage (M) –*** The coverage of the current implementation of the tool is local for Bangalore, as the specific cohort data is currently pre-loaded on the mindLAMPVis app [2]. The relative importance of the coverage is mentioned in the Methods section of the manuscript as follows:

*The clinicians clarified the local needs for such a tool. In a busy clinic in the Indian setting, mental health professionals would need quick access to meaningful and actionable digital behaviors obtained via the data generated from the mindLAMP app used by patients. A clinician-facing dashboard should be designed to bridge this gap by providing visual representations of complex high-dimensional data. These visualizations must first be interpretable by the clinicians and, optionally, by the patient. This serves as a stepping stone towards establishing digital psychiatry clinics in India, which are expected to experience high patient volume.*

***Item 16: Outcomes (M) –*** The primary outcomes of this pilot implementation have been in studying the relapse events in patients with a schizophrenia diagnosis who are using the mindLAMP app. The retrospective analysis done by the clinicians through specific case studies on relapses is provided in the Results section.

***Item 17: Lessons Learned (M) –*** The success of this work lies in the pilot implementation of the mindLAMPVis app through co-design by geographically separated and interdisciplinary teams.

The challenges remain in the sparsity of the data collected through digital phenotyping for patients with a schizophrenia diagnosis, which will affect mindLAMPVis downstream. Dimensionality reduction methods require sufficient data points for rigorous analysis.

This project was undertaken as an academic one, and hence, did not require a budget. But for sustaining development, deployment, and usage, appropriate funds must be sought from the government funding agencies to cater to public healthcare needs.

***Item 18: Unintended Consequences (NM) –*** A positive unintended consequence is that the app can cater to any other phenotypes where data is collected using digital phenotyping.

Another unintended consequence of the app itself is that comparative analysis can be done across patients, too. Currently, it is used for comparing different modalities of the data for the same patient, as mentioned in the Abstract:

*Through case examples focusing on relapse risk prediction in schizophrenia, mindLAMPVis is used to identify different visualization methods to compare different analytical results for each patient.*

A positive unintended consequence is that a few of the visualizations can be shared with patients too, e.g., hometime visualizations, even though the tool is designed to be clinician-facing.

**Section S6. Discussion**

This section includes the mandatory items related to predominantly the “Discussions” section of the manuscript.

***Item 19: Discussion (M) –*** The summary of the discussion is succinctly provided in the Abstract section as:

*mindLAMPVis is a tailored tool designed for use in India, but it can aid in identifying and comparing behavioral patterns that may indicate clinical risk for patients in any country. mindLAMPVis offers an example of how, through technical design, feedback, and real-world clinical testing, it is feasible to adapt current software tools to meet local needs and even exceed the use cases of the original technology. mindLAMPVis also successfully incorporates both active and passive digital phenotyping data.*

The future implications are provided in the Discussions section as:

*Beyond the technical results, our paper highlights how co-design is feasible through adapting existing software tools. While the technical challenges in working with the data required the computer science expertise of our team, the universal nature of coding enabled rapid progress. As the interest in digital psychiatry expands, tools that combine data analysis (data mining) and visualization, like mindLAMPVis, simplify the understanding and interpretation of digital behaviors for both clinicians and patients. This ensures that the work is more accessible and allows for more collaboration from all stakeholders.*

**Section S7. General**

Currently, there is no specific point to discuss in this section.

***Item 20: General (NM) –*** We do not anticipate any regulatory approvals required for this tool in the future, as the regulatory approvals will need to be satisfied at the data collection phase using the digital phenotyping app. The clinicians using such data will have access to the mindLAMPVis tool too.

Further regulatory approvals may be required if the data has to be shared with any third party and beyond. That is not currently considered in the design of this app, as there is no access control programmed in the current version of the software architecture of mindLAMPVis.

**References**

[1] Caroline Perrin Franck, Awa Babington-Ashaye, Damien Dietrich, Georges Bediang, Philippe Veltsos, Pramendra Prasad Gupta, Claudia Juech, Rigveda Kadam, Maxime Collin, Lucy Setian, et al. iCHECK-DH: Guidelines and checklist for the reporting on digital health implementations. Journal of Medical Internet Research, 25:e46694, 2023.

[2] Karthik Sama, Jaya Sreevalsan-Nair, Soumya Choudhary, Srilakshmi Nagendra, Preethi V Reddy, Asher Cohen, Urvakhsh Meherwan Mehta, and John Torous. mindLAMPVis: Data Visualization Portal. <https://mindlampvis.azurewebsites.net/>, 2024.

[3] Karthik Sama, Jaya Sreevalsan-Nair, Soumya Choudhary, Srilakshmi Nagendra, Preethi V Reddy, Asher Cohen, Urvakhsh Meherwan Mehta, and John Torous. MindLAMPVisDev. <https://github.com/GVCL/mindLAMPVisDev/>, 2025.

[4] John Torous, Sandra Bucci, Imogen H Bell, Lars V Kessing, Maria Faurholt-Jepsen, Pauline Whelan, Andre F Carvalho, Matcheri Keshavan, Jake Linardon, and Joseph Firth. The growing field of digital psychiatry: Current evidence and the future of apps, social media, chatbots, and virtual reality. World Psychiatry, 20(3):318–335, 2021.

[5] Dong Whi Yoo, Michael L Birnbaum, Anna R Van Meter, Asra F Ali, Elizabeth Arenare, Gregory D Abowd, and Munmun De Choudhury. Designing a clinician-facing tool for using insights from patients’ social media activity: Iterative co-design approach. JMIR Mental Health, 7(8):e16969, 2020.

[6] Aila Naderbagi, Victoria Loblay, Iqthyer Uddin Md Zahed, Mahalakshmi Ekambareshwar, Adam Poulsen, Yun JC Song, Laura Ospina-Pinillos, Michael Krausz, Mostafa Mamdouh Kamel, Ian B Hickie, et al. Cultural and Contextual Adaptation of Digital Health Interventions: Narrative Review. Journal of Medical Internet Research, 26:e55130, 2024.

[7] Hamid Mansoor, Walter Gerych, Abdulaziz Alajaji, Luke Buquicchio, Kavin Chandrasekaran, Emmanuel Agu, Elke Rundensteiner, and Angela Incollingo Rodriguez. INPHOVIS: Interactive visual analytics for smartphone-based digital phenotyping. Visual Informatics, 7(2):13–29, 2023.

[8] Anna M Langener, Gert Stulp, Nicholas C Jacobson, Andrea Costanzo, Raj R Jagesar, Martien J Kas, and Laura F Bringmann. It’s All About Timing: Exploring Different Temporal Resolutions for Analyzing Digital-Phenotyping Data. Advances in Methods and Practices in Psychological Science, 7(1):25152459231202677, 2024.

[9] Kyungmi Lee, Tim Cheongho Lee, Maria Yefimova, Sidharth Kumar, Frank Puga, Andres Azuero, Arif Kamal, Marie A Bakitas, Alexi A Wright, George Demiris, et al. Using digital phenotyping to understand health-related outcomes: A scoping review. International Journal of Medical Informatics, page 105061, 2023.

[10] Simone Schmidt and Simon D’Alfonso. Clinician perspectives on how digital phenotyping can inform client treatment. Acta Psychologica, 235:103886, 2023.

[11] Elena Rodriguez-Villa, Urvakhsh Meherwan Mehta, John Naslund, Deepak Tugnawat, Snehil Gupta, Jagadisha Thirtalli, Anant Bhan, Vikram Patel, Prabhat Kumar Chand, Abhijit Rozatkar, et al. Smartphone Health Assessment for Relapse Prevention (SHARP): a digital solution toward global mental health. BJPsych Open, 7(1):e29, 2021.

[12] Asher Cohen, John A Naslund, Sarah Chang, Srilakshmi Nagendra, Anant Bhan, Abhijit Rozatkar, Jagadisha Thirthalli, Ameya Bondre, Deepak Tugnawat, Preethi V Reddy, et al. Relapse prediction in schizophrenia with smartphone digital phenotyping during COVID-19: a prospective, three-site, two-country, longitudinal study. Schizophrenia, 9(1):6, 2023.

[13] Michael Sedlmair, Miriah Meyer, and Tamara Munzner. Design study methodology: Reflections from the trenches and the stacks. IEEE Transactions on Visualization and Computer Graphics, 18(12):2431–2440, 2012.
